# New Flavone C-Glycosides from *Scleranthus perennis* and Their Anti-Collagenase Activity

**DOI:** 10.3390/molecules26185631

**Published:** 2021-09-16

**Authors:** Katarzyna Jakimiuk, Jakub W. Strawa, Sebastian Granica, Michał Tomczyk

**Affiliations:** 1 Department of Pharmacognosy, Faculty of Pharmacy with the Division of Laboratory Medicine, Medical University of Białystok, ul. Mickiewicza 2a, 15-230 Białystok, Poland; katarzyna.jakimiuk@umb.edu.pl (K.J.); jakub.strawa@umb.edu.pl (J.W.S.); 2 Microbiota Lab, Center for Preclinical Studies, Department of Pharmacognosy and Molecular Basis of Phyto therapy, Faculty of Pharmacy, Medical University of Warsaw, ul. Banacha 1, 02-097 Warsaw, Poland; sgranica@wum.edu.pl

**Keywords:** *Scleranthus perennis*, Caryophyllaceae, flavonoids, isolation, scleranthoside, *C*-glycosides, collagenase

## Abstract

Three new flavone glycosides, one known flavone glycoside, and the phenolic derivative apiopaenonside were isolated and identified from the ethyl acetate fraction of the aerial parts of *Scleranthus perennis*. The planar structures were elucidated through extensive analysis of UV-Vis, IR, and ^1^H NMR and ^13^C NMR spectral data, including the 2D techniques COSY, HSQC, and HMBC, as well as ESI mass spectrometry. The isolated compounds were established as 5,7,3′-trihydroxy-4′-acetoxyflavone-8-*C*-*β*-d-xylopyranoside-2′′-*O*-glucoside (**1**), 5,7,3′-trihydroxy-4′-methoxyflavone-8-*C-β*-d-xylopyranoside-2′′-*O*-glucoside (**2**), 5,7-dihydroxy-3′-methoxy-4′-acetoxyflavone-8-*C*-*β*-d-xylopyranoside-2′′-*O*-glucoside (**3**), 5,7-dihydroxy-3′-methoxy-4′-acetoxyflavone-8-*C*-*β*-d-xylopyranoside-2′′-*O*-(4′′′-acetoxy)-glucoside (**4**), and apiopaenonside (**5**). Moreover, all isolated compounds were evaluated for anti-collagenase activity. All compounds exhibited moderate inhibitory activity with IC_50_ values ranging from 36.06 to 70.24 µM.

## 1. Introduction

The genus *Scleranthus* L. (Caryophyllaceae) comprises 11 named species divided into two sections, *Scleranthus* and *Mniarum*. *Scleranthus* includes three endemic species to Europe, Western Asia, and North Africa (*S. annuus*, *S. perennis*, *S. uncinatus*), as well as three Australian endemic species (*S. diander*, *S. pungens*, *S. minusculus*). The plants of *Scleranthus* are widespread perennial herbs occupying mainly dry, sandy, or disturbed habitats [1,2]. Previous phytochemical studies have revealed the presence of flavonoids in *S. uncinatus* [3,4]. Furthermore, phenolic acids have been isolated from *S. perennis* water/alcoholic extracts, while sapogenins, tannins, and sterols were identified from butanol extracts [5,6]. *S. annuus* water/alcoholic extracts are a source of phenolic acids and flavonoids [7]. *S. perennis* has not been well studied, and thus its phytochemical and pharmacological data are scarce. In folk medicine, this plant has been used for veterinary purposes as a remedy for animals that display a fluctuating temperament [8].

In our continuing phytochemical investigation of this plant, we isolated five compounds. According to high-performance liquid chromatography coupled with diode-array detection and mass spectrometry (UHPLC-DAD-MS) analysis and the UV-Vis spectra, four of the obtained structures were classified as derivatives of flavones [9,10] and one was identified as a paeonol derivative. In the present work, we performed investigations and identified *C*-glycosylated luteolin derivatives and apiopaenonside in an ethyl acetate fraction of *S. perennis.* For these compounds, ^1^H NMR and ^13^C NMR analyses, including the 2D techniques COSY, HMBC, and HSQC, as well as UV-Vis, IR, HR-ESI-MS, product ion scan, and acid hydrolysis, were performed. To the best of our knowledge, three of the isolated compounds are new chemical structures found in the plant kingdom. The present communication addresses their isolation and structural elucidation as well as bioactivity evaluation of these compounds.

## 2. Results and Discussion

**Figure 1:**
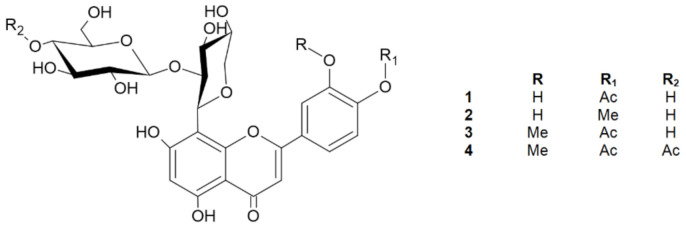
Chemical structures of compounds **1–4**.

The preliminary LC-MS screening of the ethyl acetate fraction from the dried aerial parts of *S. perennis* showed the presence of polyphenol derivatives [9]. Thus, the ethyl acetate fraction was separated multiple times by preparative, providing four flavone derivatives (**1**–**4**) and one phenolic derivative (**5**) (Figure 1).

### 2.1. 5,7,3′-Trihydroxy-4′-acetoxyflavone-8-C-β-d-xylopyranoside-2″-O-glucoside (**1**)

**Figure 2:**
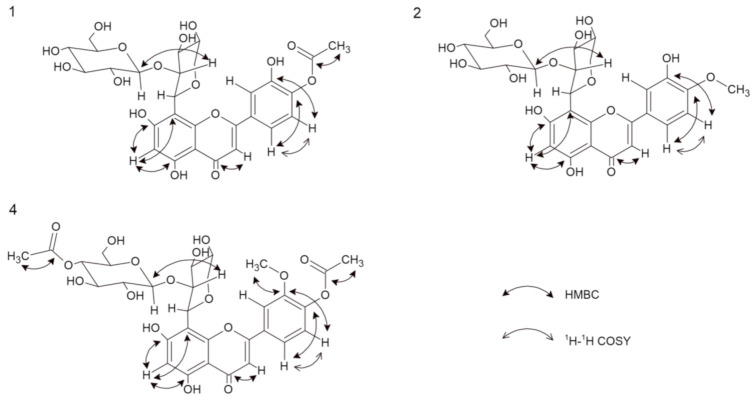
Important ^1^H-^1^H COSY and HMBC correlation of compounds **1**, **2**, and **4**.

Compound **1** was obtained as yellow amorphous powder. Based on the HRESIMS ion peak at *m/z* 623 [M + H]^+^, the molecular formula of C_28_H_30_O_16_ was determined. The UV spectrum exhibited absorption maxima at 248 and 305 nm, which is typical of flavones. A free C7 hydroxyl group was confirmed by a bathochromic shift of 6 nm (in the presence of sodium acetate (NaOAc)), and a free C5 hydroxyl group was indicated by a bathochromic shift of 41 nm (in the presence of aluminum chloride (AlCl_3_)). Furthermore, a bathochromic shift of 4 nm (in the presence of sodium methoxide (NaOMe)) indicated that C4′ is substituted [11]. The ^1^H NMR spectrum showed one proton singlet at *δ* 6.20 characterizing a trisubstituted A-ring, while the absence of aromatic methine carbon signals in the range of 90–96 ppm suggested that C8 was substituted. Based on the HMBC data, the proton at *δ* 6.19 showed correlations with C5 and C8; thus, this signal was assigned to C6 [3,12]. Detailed analysis of the ^13^C NMR data led to the assignment of the carbons in the B-ring. The signals at *δ* 7.42 (1H, d, *J* = 8.28 Hz) and *δ* 6.92 (1H, d, *J* = 8.28 Hz) were assigned to H-C5′ and H-C6′, respectively, and these assignments were confirmed by COSY correlations. Moreover, the carbon signals at *δ* 150.90 (C4′) and *δ* 146.92 (C3′) display ortho coupling, as found in 3′,4′-oxygenated flavonoids [13]. The presence of the unsaturated bond was shown by the *δ* 184.17 signal in the ^13^C NMR spectrum, which corresponds to C4 of the C-ring [3,14]. From the HMBC analysis, the C4 carbon signal was linked with the proton signal at *δ* 6.51 assigned as H-C3. The presence of an acetoxy group in the structure at C4′ was revealed by the chemical shift of the -CH_3_ group in the ^1^H NMR spectrum at *δ* 1.98 (s, 3H), as well as in the ^13^C NMR spectrum for an acetoxyl carbonyl carbon at *δ* 172.98 and an acetoxyl methyl carbon at *δ* 20.79 [3,13]. This conclusion was further supported by HSQC and HMBC correlations. The ^1^H NMR spectrum revealed the two anomeric protons at *δ* 5.08 (1H, d, *J* = 9.54 Hz) and *δ* 4.29 (1H, d, *J* = 7.78 Hz), which are characteristic of two sugars with *β*-configurations [15]. Based on the HMBC and HSQC correlations, the anomeric carbons appeared at *δ* 74.90 and *δ* 105.90. Extensive analysis of the ^1^H NMR, ^13^C NMR, DEPT, and 2D NMR spectral data, including COSY, HSQC, and HMBC, found the individual saccharide chemical shifts that are shown in Table 1 and Figure 2 [13]. According to the obtained data, one of the saccharides was *β*-d-glucose, and the second was β-d-xylopyranoside [4,14,16]. One sugar (terminal) was also analyzed by thin-layer chromatography (TLC) after acid hydrolysis of compound **1** and was determined to be glucose. Interference between H-C1′′′ and H-C2′′, as well as H-C1′′ and C8 from the HMBC data, suggests that the sugars are linked by Glc(1′′′→2′′)Xyl bonds. Moreover, the type of bonds and substitutions were confirmed based on triple-quadrupole MS fragmentation. The ion fragmentation pattern of flavonoids shows a retro-Diels–Alder reshuffling in the C-ring with the loss of neutral molecules of water, saccharides, and methyl and carbonyl groups [17]. Compound **1** showed a predominant molecular ion at *m/z* 621 [M − H]^−^. Other fragments were as follows (CE = −20 eV): 399 [M-H-glucose-OAc], which indicated the presence of the neutral loss of hexose and an acetoxy group from the structure; 309 [M-H-glucose-OAc-part of xylose]; and 175 [M-H-glucose-OAc-part of xylose-C_8_H_6_O-OH]. The loss of C_8_H_6_O suggests that C3 (of the C-ring) is not substituted with a hydroxyl group. On the other hand, the loss of this moiety may indicate a hydroxyl group at C3′ (of the B-ring) [17]. Moreover, the IR spectrum showed typical signals for O–H (V_max_ 3462), C–H (V_max_ 2950), C=O (V_max_ 1716), and C=C (V_max_ 1616) [17]. Therefore, the new chemical structure from plants, 5,7,3′-trihydroxy-4′-acetoxyflavone-8-*C*-*β*-d-xylopyranoside-2′′-*O*-glucoside (Figure 1) named scleranthoside A, was definitively established.

### 2.2. 5,7,3′-Trihydroxy-4′-methoxyflavone-8-C-β-d-xylopyranoside-2″-O-glucoside (**2**)

Compound **2** was also isolated as a yellow amorphous powder. Its molecular formula of C_27_H_30_O_15_ was established based on the positive HRESIMS ion peak at *m/z* 595 [M + H]^+^. Detailed analysis of the ^1^H NMR and ^13^C NMR data (Table 1), including the 2D techniques COSY, HMBC, and HSQC, of compound **2** showed that its planar structure and sugar side chain were identical to those of compound **1**, but some slight differences in chemical shifts were observed, mainly concerning the C4′ moiety. The ^1^H NMR spectrum showed a signal at *δ* 4.01 (s, 3H) corresponding to the -CH_3_ instead of the acetoxy group observed in compound **1**. Furthermore, the ^13^C NMR data confirmed the methoxyl group at *δ* 56.84 [13], which was assigned to the 4′ carbon in the C-ring. The site of methylation was further supported by the HMBC data observed for C4′ (*δ* 151.88) with a methyl group (*δ* 4.01) (Figure 2). Compound **2** showed a major molecular ion at *m/z* 593 [M − H]^−^ (CE = −20 eV). In the negative ESI mass spectrum, the fragment at *m/z* 413 [M-glucose-H] corresponded to the subsequent loss of the hexose moiety at the terminal position, and the neutral loss at *m/z* 398 [M-glucose-H-CH_3_] indicated the loss of a -CH_3_ moiety. Additionally, the IR spectrum showed characteristic signals for O–H (V_max_ 3420), C–H (V_max_ 2903), C=O (V_max_ 1716), and C=C (V_max_ 1654) [18]. Thus, the structure of **2**, which is a new natural product, was established as 5,7,3′-trihydroxy-4′-methoxyflavone-8-*C*-*β*-d-xylopyranoside-2′′-*O*-glucoside and named scleranthoside B (Figure 1).

### 2.3. 5,7-Dihydroxy-3′-methoxy-4′-acetoxyflavone-8-C-β-d-xylopyranoside-2″-O-glucoside (**3**)

Compound **3** showed an [M + H]^+^ ion at *m/z* 637 in its HRESIMS spectrum, corresponding to the molecular formula C_29_H_32_O_16_. This structure exhibited flavone and sugar skeletons similar to those of compounds **1** and **2** except for the signals at the 3′ and 4′ carbons of the C-ring. The presence of a methoxyl group in the molecule was indicated by a peak in the ^1^H NMR spectrum at *δ* 4.01, which appeared as a singlet and integrated to 3H, and in the ^13^C NMR spectrum it appeared as one signal at *δ* 56.71. This methoxyl group was placed on carbon 3′ (*δ* 149.50) based on the HMBC correlations of this group with C3′. In addition, from long-range COSY connectivities, the position of the methoxyl group on the B-ring was confirmed due to the cross-peaks from H2′ (*δ* 7.65). Furthermore, in the ^1^H NMR spectrum, we observed a chemical shift for the –CH_3_ of the acetoxyl group (*δ* 1.94, s, 3H), and in the ^13^C NMR spectrum, signals for an acetoxyl carbonyl carbon at *δ* 172.93 and an acetoxyl methyl carbon at *δ* 20.73 were observed, which, according to the HMBC and COSY data, were assigned to the C4′ position. Compound **3** produced a minor protonated ion at *m/z* 635 [M − H]^−^ in the negative mode. In addition, the fragment appearing at 413 [M-H-glucose-OAc] (CE = −20 eV) indicated the presence of the neutral loss of hexose and an acetoxy group from the structure. The 1D and 2D NMR signals of compound **3** are consistent with literature data [3]. Based on the above observations, the dominant structure in the EtOAc fraction, compound **3**, was established as 5,7-dihydroxy-3′-methoxy-4′-acetoxyflavone-8-*C*-*β*-d-xylopyranoside-2′′-*O*-glucoside (Figure 1).

### 2.4. 5,7-Dihydroxy-3′-methoxy-4′-acetoxyflavone-8-C-β-d-xylopyranoside-2″-O-(4″′-acetoxy)-glucoside (**4**)

Compound **4**, a yellow amorphous powder, exhibited a predominant ion peak at *m/z* 679 [M + H]^+^ in positive mode by HRESIMS, corresponding to the molecular formula C_31_H_34_O_17_. This was corroborated by the ^13^C NMR data, which showed signals for 31 carbons (Table 1). Comparison of the NMR data of **3** and **4** suggested that their structures were highly similar. The signals in the ^13^C NMR spectrum revealed two acetoxy groups at *δ* 19.08 and *δ* 19.58. The significant difference between the structures of **3** and **4** was the additional acetoxy group at C4′′′ in the sugar chain. The presence of this group was further verified by the HMBC correlation of C4′′′ (*δ* 69.68) with the methyl acetoxyl carbon, which resonates at *δ* 19.58 (Figure 2). Additionally, from the HSQC data, carbon 4′′′ of the glucose chain was linked with the protons of the methyl acetoxyl moiety (*δ* 1.95). A product ion scan revealed that compound **4** produced a minor protonated ion at *m/z* 677 [M − H]^−^ in the negative mode. In addition, the fragment appearing at *m/z* 413 [M-H-glucose-OAc] (CE = −20 eV) indicated the presence of the neutral loss of hexose and an acetoxyl group from the structure. The high-energy ion fragmentation pathway (CE = −70 eV) showed *m/z* 59, which may indicate an ion for the second acetoxyl group. Moreover, the IR spectrum showed typical signals for O–H (V_max_ 3420), C–H (V_max_ 2899), C=O (V_max_ 1732), and C=C (V_max_ 1656) [18]. Hence, the structure of **4** was identified as 5,7-dihydroxy-3′-methoxy-4′-acetoxyflavone-8-*C*-*β*-d-xylopyranoside-2′′-*O*-(4′′′-acetoxy)-glucoside and this compound was given the trivial name scleranthoside C (Figure 1).

### 2.5. Apiopaenonside (**5**)

**Figure 3:**
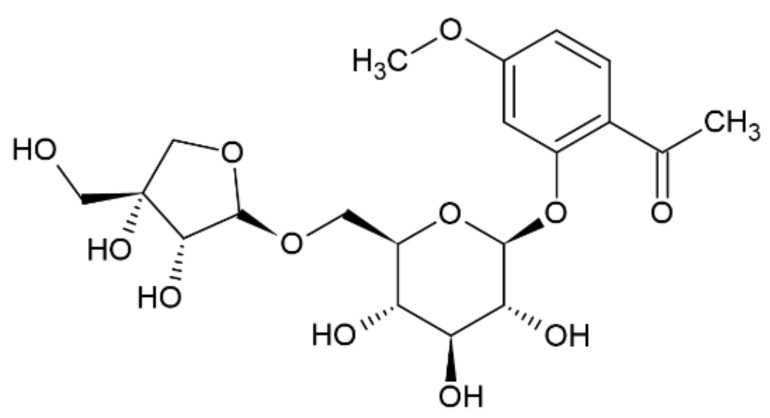
Chemical structure of compound **5**.

Compound **5** was obtained as a yellow amorphous powder. The molecular formula C_20_H_28_O_12_ was deduced from its positive mode HRESIMS, which showed a molecular ion peak [M + H]^+^ at *m/z* 460. In the ^1^H NMR spectrum, the peaks between *δ* 6.50 and *δ* 8.00 correspond to hydrogen atoms in the aromatic ring. Furthermore, the signals at *δ* 7.68, *δ* 7.58, and *δ* 7.23 indicate the presence of substitutions on C1, C2, and C4, respectively [13]. Furthermore, HSQC correlations verified the *δ* 124.6 (C1), 112.4 (C2), and 116 (C4) signals in the ^13^C spectrum. In addition, a singlet at *δ* 2.58 indicated the presence of a CH_2_ group, where the hydrogens do not interact with any other protons. Based on chromatographic, spectral analysis and the literature data, compound **5** was established as apiopaenonside (Figure 3), which was previously isolated from *Paeonia suffruticosa* [19,20].

Compounds **1**–**5** were evaluated for their anti-collagenase activity. As shown in Table 2, the highest inhibitory activity was possessed by 5,7-dihydroxy-3′-methoxy-4′-acetoxyflavone-8-*C*-*β*-d-xylopyranoside-2′′-*O*-(4′′′-acetoxy)-glucoside (**4**) with an IC_50_ (median inhibitory concentration) value of 36.06 µM, compared to epigallocatechin gallate (34.32 µM).

## 3. Materials and Methods

### 3.1. General Experimental Procedures

Acetonitrile Optima (ACN), was purchased from Fisher Chemical (Loughborough, UK). Ultra-pure water (UPW) was obtained using the POLWATER DL3-100 system (Kraków, Poland). Petrol, chloroform (CHCl_3_), ethyl acetate (EtOAc), diethyl ether (Et_2_O), *n*-butanol (BuOH), formic acid (FA), hydrochloric acid (HCl), acetic acid (AcOH), dimethyl sulfoxide (DMSO), ammonia solution 25% (NH_4_OH), ethanol (EtOH), and methanol (MeOH) were purchased from POCH (Gliwice, Poland). Natural product reagent A (NA) was purchased from Carl Roth (Karlsruhe, Germany). To prepare aniline phthalate, phthalic acid was purchased from Sigma-Aldrich (Poole, Great Britain) and aniline from Chempur (Piekary Śląskie, Poland). Epigallocatechin gallate (EGCG) as a positive control was obtained from Cayman Chemical (Ann Arbor, MI, USA). Collagenase from *Clostridium histolyticum* (C0130), sodium chloride (NaCl), calcium chloride (CaCl_2_), and N-[3-(2-Furyl)acryloyl]-leu-gly-Pro-Ala (FALGPA) were obtained from Sigma-Aldrich (Poole, UK). Monosaccharides for TLC analysis of the hydrolysis products (glucose, xylose, rhamnose) were purchased from Merck KGaA (Darmstadt, Germany), and uronic acids (glucuronide acid, galacturonide acid) were purchased from Cayman Chemical (Ann Arbor, MI, USA). Luteolin (purity > 96%) was isolated from the inflorescences of *Arctium tomentosum* [21]. Sephadex LH-20 was provided by GE Healthcare Bio-Sciences AB (Uppsala, Sweden). TLC plates coated with silica gel (105554) and microcrystalline cellulose (105716) were purchased from Merck KGaA (Darmstadt, Germany). LC-MS analyses were conducted using an Agilent Technologies 1260 Infinity chromatography system connected to a 6230 time-of-flight (TOF) mass spectrometer (Santa Clara, CA, USA). Preparative HPLC analyses were carried out on a Shimadzu instrument (Columbia, MD, USA) with LC20-AP pumps, an SPD-10ATvp detector, an LC-10AF autosampler, and an FRC-10A fraction collector. Liquid chromatography triple-quadrupole mass spectrometry was performed on a Shimadzu LC-MS 8050 Triple Quad spectrometer coupled with a Nexera LC system (Kyoto, Japan) consisting of an SCL-40 system controller, a DGU-405 degasser, an LC-40D xR pump, a CTO-40S column oven, and an SIL-40C xR autosampler. UV spectra were measured with an Analytic Jena SPECORD 200 Plus instrument (Jena, Germany). Melting points were obtained using a BUCHI 535. Column chromatography (CC) was performed with a Sephadex LH-20 column. IR spectra were recorded on a Perkin Elmer FT-IR spectrometer spectrum-2000 (Waltham, MA, USA) using potassium bromide (KBr) pellets. NMR spectra were recorded on a Thermo Fisher Scientific Bruker Avance II 400 spectrometer (Waltham, MA, USA) at 400 MHz in deuterated methanol (CD_3_OD). Optical rotations were measured with JASCO P-2000 (Tokyo, Japan). Bioassay was performed on BioTek Instruments microplate spectrophotometer EPOCH 2 (Oxfordshire, UK).

### 3.2. Plant Material

The aerial parts of *Scleranthus perennis* were collected between August and September 2018 in the Bialystok area (53°06′39.0′′ N 23°07′13.4′′ E) in Poland. The plant was authenticated based on its morphological characteristics by one of the authors (MT) according to Rutkowski [22]. A plant voucher specimen (No. SP-18041) was deposited in the Herbarium of the Department of Pharmacognosy at the Medical University of Białystok, Poland.

### 3.3. Extraction and Isolation

The dried and powdered aerial parts of *S. perennis* (1100 g) were partitioned successively with petrol, chloroform, and methanol. The MeOH extract was concentrated to dryness under vacuum at a controlled temperature (30 ± 2 °C) and subjected to lyophilization until a constant weight was obtained (108 g). The extract was dissolved in MeOH (110 g) and subjected to CC (85 cm × 5 cm) on a Sephadex LH-20 column. The column was eluted with MeOH to give 33 fractions (~50 mL each). Based on TLC silica gel plate developed with EtOAc:H_2_O:FA at a ratio of 18:1:1 and derivatized with 1% NA and LC-MS analyses (UPW:ACN 5→95), all fractions were pooled into five main fractions (F1–F5). The aqueous residue of F3 was fractionated by liquid−liquid extraction with Et_2_O, EtOAc, and finally *n*-BuOH. The combined layers were evaporated and purified. LC-MS analysis of the EtOAc fraction showed compounds that could be classified as derivatives of flavonoids. The EtOAc fraction (2 g) was dissolved in DMSO, and part of this fraction (2.5 g) was separated by preparative HPLC (0–35 min, 0%–7% UPW-ACN, 20 mL/min) to obtain compound **1** (11.75 mg), compound **2** (13.8 mg), compound **3** (385.56 mg), compound **4** (31.3 mg), and compound **5** (8.69 mg). The purified compounds were identified based on chromatographical products of acid hydrolysis (TLC; Rf: 0.55 corresponds to glucose standard) and the recorded ^1^H, ^13^C, COSY, HSQC, and HMBC spectra in CD_3_OD, as well as MS, IR, and UV spectra, and the product ion scan.

### 3.4. Acid Hydrolysis

Approximately 3 mg of compounds **1**–**4** was refluxed in 2N HCl (5 mL) for 2 h. The aglycones from the post-hydrolyzed solution (PHS) were extracted with Et_2_O and identified by TLC with standard. The TLC plate was developed with 30:3:10 solvent system (HCl:AcOH:H_2_O). TLC analysis of monosaccharides residues was conducted by spotting standards, the PHS water layer, and developing with a solvent system of 20:1:4 (EtOH:NH_4_OH:H_2_O). TLC chromatograms were derivatized using freshly prepared aniline phthalate, heating, and comparing with Rf values of standards.

### 3.5. Isolates

5,7,3′-trihydroxy-4′-acetoxyflavone-8-*C*-*β*-d-xylopyranoside-2′′-*O*-glucoside (**1**): yellow amorphous powder (mp.: 188.5–189.0 °C); [α]_D_ +21.1 (DMSO; c 0.1); HPLC rt, 48.1 min; HRESIMS *m/z* = 623.18 [M + H]^+^ (calculated for C_28_H_30_O_16_); UV λ_max_ nm: 254, 305; +NaOMe: 253, 309; +AlCl_3_: 254, 346; +NaOAc: 254, 311; +H_3_BO_3_: 248, 320; IR *V*_max_ (KBr) 3462 (s, O–H), 2950 (s, C–H), 1716 (s, C=O), 1616 (s, C=C); NMR spectral data, see Table 1 and Figures S1–S9 in the Supplementary Materials.

5,7,3′-trihydroxy-4′-methoxyflavone-8-*C*-*β*-d-xylopyranoside-2′′-*O*-glucoside (**2**): yellow amorphous powder (mp.: 178.5–179.2 °C); [α]_D_ -9.2 (DMSO; c 0.1) HPLC rt, 51.2 min; HRESIMS *m/z* = 595.18 [M + H]^+^ (calculated for C_27_H_30_O_15_); UV λ_max_ nm: 248, 288; +NaOMe: 252, 302; +AlCl_3_: 251, 316; +NaOAc: 257, 303; +H_3_BO_3_: 250, 298; IR *V*_max_ (KBr) 3420 (s, O–H), 2903 (s, C–H), 1716 (s, C=O), 1654 (s, C=C); NMR spectral data, see Table 1 and Figures S10–S18 in the Supplementary Materials.

5,7-dihydroxy-3′-methoxy-4′-acetoxyflavone-8-*C*-*β*-d-xyloside-2′′-*O*-glucoside (**3**): yellow amorphous powder; HPLC rt, 55.2 min; HRESIMS *m/z* = 637.18 [M + H]^+^ (calculated for C_30_H_31_O_16_); UV λ_max_ nm: 253, 271, 347; +NaOMe: 266, 404; +AlCl_3_: 277, 392; +NaOAc: 273, 352; +H_3_BO_3_: 212, 346; NMR spectral data, see [2] and Figures S19–S23 in the Supplementary Materials.

5,7-dihydroxy-3′-methoxy-4′-acetoxyflavone-8-*C*-*β*-d-xylopyranoside-2′′-*O*-(4′′′-acetoxy)-glucoside (**4**): yellow amorphous powder (mp.: 164.6–166.2 °C); [α]_D_ +10.6 (DMSO; c 0.5); HPLC rt, 60.4 min; HRESIMS *m/z* = 679.22 [M + H]^+^ (calculated for C_31_H_34_O_17_); UV λ_max_ nm: 253, 287; +NaOMe: 251, 304; +AlCl_3_: 249, 320; +NaOAc: 259, 304; +H_3_BO_3_: 263, 301; IR *V*_max_ (KBr) 3420 (s, O–H), 2899 (s, C–H), 1732 (s, C=O), 1656 (s, C=C); NMR spectral data, see Table 1 and Figures S24–S32 in the Supplementary Materials.

Apiopaenonside (**5**): yellow amorphous powder; HPLC rt, 18.6 min; HRESIMS *m/z* = 460 [M + H]^+^ (calculated for C_20_H_28_O_12_); UV λ_max_, see [19], NMR spectral data, see [20].

### 3.6. In Vitro Collagenase Inhibition Assay

The previous spectrophotometric procedure was modified and subsequently employed to determine the anti-collagenase activity of the isolated compounds [23]. This assay was performed in 50 mM Tricine buffer (pH = 7.5; 400 mM NaCl, 10 mM CaCl_2_). The mixed solution included 25 µL of 0.1 U/mL collagenase from *Clostridium histolyticum*, 25 µL Tricine buffer, and 25 µL of various levels of the sample were incubated at 37 °C for 20 min. After incubation, 75 µL of 0.8 mM FALGPA substrate was present. Then, absorbance was measured at 335 nm wavelength. Negative control was performed using Tricine buffer instead of sample and positive control was conducted with EGCG.

The percentage inhibition for assay was calculated by:

Enzyme inhibition activity (%) = [1 − (C/S)] × 100%

where C is the negative control and S is the sample.

### 3.7. Statistical Analysis

All results are expressed as the mean ± standard deviation (SD) and analyses were performed in triplicate. Significant statistical analysis was performed using GraphPad Prisma 9 software (GraphPad Software, San Diego, CA, USA). Statistical differences were assessed using one-way ANOVA.

## 4. Conclusions

The occurrence of **1**–**4** constitutes this as the first report of flavone *C*-glycosides from the *Scleranthus perennis*. Compound **5**, a derivative of paeonol, was also newly found in the Caryophyllaceae family. Furthermore, to the best of our knowledge, compounds **1**, **2**, and **4** are new chemical structures occurring in the plant kingdom. Their discovery not only extends the structural and chemical diversity of phenolic compound, but also underlines the potential source for bioactive natural products. Further investigations on their biological activities are in progress.

## Figures and Tables

**Table 1:** ^1^H and ^13^C spectral data of **1**, **2**, and **4** (CD_3_OD, 400 Hz, δ in ppm, *J* in Hz).

C No.	1		2		4	
	δ_C_	δ_H_	δ_C_	δ_H_	δ_C_	δ_H_
2	166.36	-	166.21	-	164.80	-
3	104.46	6.51, *s*	104.24	6.61, *s*	104.01	6.60, *s*
4	184.17	-	184.25	-	182.79	-
5	162.62	-	162.82	-	161.27	-
6	100.86	6.19, *s*	100.78	6.27, *s*	99.47	6.21, *s*
7	164.55	-	164.56	-	163.27	-
8	103.92	-	104.38	-	102.09	-
9	156.89	-	156.95	-	155.51	-
10	105.36	-	105.48	-	106.32	-
-OMe	-	-	56.69	4.01 (*s*, 3H)	55.31	4.03 (*s*, 3H)
-OAc	172.98	-	-	-	170.29	-
	20.79	1.98, *s*	-	-	19.08	1.93, *s*
1′	124.16	-	124.13	-	122.81	-
2′	114.50	7.68, *s*	111.03	7.61, *s*	109.71	7.66, *s*
3′	146.92	-	149.45	-	148.09	-
4′	150.90	-	151.88	-	150.52	-
5′	116.85	6.92 (*d*, *J* = 8.28)	117.07	6.96 (*d*, *J* = 8.28)	115.41	6.96 (*d*, *J* = 8.28)
6′	120.78	7.42 (*d*, *J* = 8.28)	121.84	7.50 (*d*, *J* = 8.28)	120.41	7.51 (*d*, *J* = 8.28)
1′′	74.90	5.08 (*d*, 1H, *J* = 9.54)	76.02	5.10 (*d*, 1H, *J* = 9.54)	71.40	5.14 (*d*, 1H, *J* = 9.54)
2′′	81.66	3.99	81.36	3.85	81.52	3.87
3′′	75.95	3.76	77.11	3.76	73.34	3.85
4′′	70.17	4.20	70.22	3.89	68.60	3.91
5′′	71.92	4.06	72.09	3.87	70.58	3.90
1′′′	105.89	4.29 (*d*, 1H, *J* = 7.78)	105.98	4.31 (*d*, 1H, *J* = 7.78)	104.88	4.34 (*d*, 1H, *J* = 7.78)
2′′′	74.90	2.91	76.02	3.15	71.40	3.33
3′′′	77.76	3.01	77.98	3.19	74.64	3.37
4′′′	70.67	2.93	71.82	3.17	69.68	3.23
5′′′	75.57	3.15	75.88	3.27	71.40	3.38
6′′	64.58	3.17	63.12	3.37	61.60	3.41
-OAc	-	-	-	-	171.26	-
	-	-	-	-	19.58	1.96, *s*

**Table 2:** Anti-collagenase activity of compounds **1**–**5** and their respective IC_50_ values.

Compounds	IC_50_ ^a^ (µM)
**1**	70.24 ± 1.37
**2**	64.86 ± 1.08
**3**	48.28 ± 1.05
**4**	36.06 ± 0.78
**5**	>125
EGCG ^b^	34.32 ± 0.21

^a^ All data are represented as the mean of IC_50_ values with standard deviation from triplicate measurement; ^b^ Positive control.

## Data Availability

Data are contained within the article and Supplementary Materials.

## Supplementary Materials

The following are available online, Figure S1: Product ion scan in positive mode of **1**; Figure S2: Product ion scan in negative mode of **1**; Figure S3: UV spectrum of **1**; Figure S4: IR spectrum of **1** in KBr; Figure S5: ^1^H NMR spectrum (400 MHz) of **1** in CD_3_OD; Figure S6: ^13^C NMR spectrum (400 MHz) of **1** in CD_3_OD; Figure S7: ^1^H-^1^H COSY spectrum of **1** in CD_3_OD; Figure S8: HSQC spectrum of **1** in CD_3_OD; Figure S9: HMBC spectrum of **1** in CD_3_OD; Figure S10: Product ion scan in positive mode of **2**; Figure S11: Product ion scan in negative mode of **2**; Figure S12: UV spectrum of **2**; Figure S13: IR spectrum of **2** in KBr; Figure S14: ^1^H NMR spectrum (400 MHz) of **2** in CD_3_OD; Figure S15: ^13^C NMR spectrum (400 MHz) of **2** in CD_3_OD; Figure S16: ^1^H-^1^H COSY spectrum of **2** in CD_3_OD; Figure S17: HSQC spectrum of **2** in CD_3_OD; Figure S18: HMBC spectrum of **2** in CD_3_OD; Figure S19: ^1^H NMR spectrum (400 MHz) of **3** in CD_3_OD; Figure S20: ^13^C NMR spectrum (400 MHz) of **3** in CD_3_OD; Figure S21: ^1^H-^1^H COSY spectrum of **3** in CD_3_OD; Figure S22: HSQC spectrum of **3** in CD_3_OD; Figure S23: HMBC spectrum of **3** in CD_3_OD; Figure S24: Product ion scan in positive mode of **4**; Figure S25: Product ion scan in negative mode of **4**; Figure S26: UV spectrum of **4**; Figure S27: IR spectrum of **4** in KBr; Figure S28: ^1^H NMR spectrum (400 MHz) of **4** in CD_3_OD; Figure S29: ^13^C NMR spectrum (400 MHz) of **4** in CD_3_OD; Figure S30: ^1^H-^1^H COSY spectrum of **4** in CD_3_OD; Figure S31: HSQC spectrum of **4** in CD_3_OD; Figure S32: HMBC spectrum of **4** in CD_3_OD.

## Sample Availability

Samples of the compounds **1**–**5** are available from the authors.

## Abbreviations

NMR	nuclear magnetic resonance
COSY	correlation spectroscopy
HSQC	heteronuclear single quantum coherence
HMBC	heteronuclear multiple bond correlation
DEPT	distortionless enhancement by polarization transfer
IR	infrared spectroscopy
UV	ultraviolet radiation
UV-Vis	ultraviolet-visible spectroscopy
ESI	electrospray ionization
IC_50_	median inhibitory concentration
MS	mass spectrometer
LC-MS	liquid chromatography–mass spectrometry
HPLC	high-performance liquid chromatography
HRESIMS	high-resolution electrospray ionization mass spectrometry
TLC	thin-layer chromatography
Glc	glucose
Xyl	xylose
NaOAc	sodium acetate
NaOMe	sodium methoxide
AlCl_3_	aluminum chloride
NaCl	sodium chloride
CaCl_2_	calcium chloride
FALGPA	N-[3-(2-Furyl)acryloyl]-leu-gly-Pro-Ala
EGCG	epigallocatechin gallate
TOF	time-of-flight
CD_3_OD	deuterated methanol
DMSO	dimethyl sulfoxide
CC	column chromatography
MeOH	methanol
Et_2_O	diethyl ether
EtOAc	ethyl acetate
*n*-BuOH	*n*-butanol
UPW	ultra-pure water
ACN	acetonitrile
mp	melting point
SD	standard deviation
ANOVA	analysis of variance
PHS	post-hydrolyzed solution
