# Supplementary material for: New Flavone C-Glycosides from Scleranthus perennis and Their Anti-Collagenase Activity
Source: Molecules. 2021 Sep 16;26(18):5631. doi: 10.3390/molecules26185631 (PMC8468783; doi:10.3390/molecules26185631)

molecules

MDPI

Supporting Materials

New Flavone C-Glycosides from *Scleranthus perennis* and Their Anti-Collagenase Activity

Katarzyna Jakimiuk [1], Jakub W. Strawa [1], Sebastian Granica [2], and Michał Tomczyk [1,*]

[1] Department of Pharmacognosy, Faculty of Pharmacy with the Division of Laboratory Medicine, Medical University of Białystok, ul. Mickiewicza 2a, 15-230 Białystok, Poland; katarzyna.jakimiuk@umb.edu.pl (K.J.), jakub.strawa@umb.edu.pl (J.W.S.); michal.tomczyk@umb.edu.pl (M.T.)

[2] Microbiota Lab, Center for Preclinical Studies, Department of Pharmacognosy and Molecular Basis of Phyto therapy, Faculty of Pharmacy, Medical University of Warsaw, ul. Banacha 1, 02-097 Warsaw, Poland; sgranica@wum.edu.pl (S.G.)

* Correspondence: michal.tomczyk@umb.edu.pl; Tel.: +48 85 748 56 94

Contents

Fig. 1S. Product ion scan in positive mode of **1**
Fig. 2S. Product ion scan in negative mode of **1**
Fig. 3S. UV spectrum of **1**
Fig. 4S. IR spectrum of **1** in KBr
Fig. 5S. ^{1}H NMR spectrum (400 MHz) of **1** in CD_3OD
Fig. 6S. ^{13}C NMR spectrum (400 MHz) of **1** in CD_3OD
Fig. 7S. ^{1}H-^{1}H COSY spectrum of **1** in CD_3OD
Fig. 8S. HSQC spectrum of **1** in CD_3OD
Fig. 9S. HMBC spectrum of **1** in CD_3OD
Fig. 10S. Product ion scan in positive mode of **2**
Fig. 11S. Product ion scan in negative mode of **2**
Fig. 12S. UV spectrum of **2**
Fig. 13S. IR spectrum of **2** in KBr
Fig. 14S. ^{1}H NMR spectrum (400 MHz) of **2** in CD_3OD
Fig. 15S. ^{13}C NMR spectrum (400 MHz) of **2** in CD_3OD
Fig. 16S. ^{1}H-^{1}H COSY spectrum of **2** in CD_3OD
Fig. 17S. HSQC spectrum of **2** in CD_3OD
Fig. 18S. HMBC spectrum of **2** in CD_3OD
Fig. 19S. ^{1}H NMR spectrum (400 MHz) of **3** in CD_3OD
Fig. 20S. ^{13}C NMR spectrum (400 MHz) of **3** in CD_3OD
Fig. 21S. ^{1}H-^{1}H COSY spectrum of **3** in CD_3OD
Fig. 22S. HSQC spectrum of **3** in CD_3OD
Fig. 23S. HMBC spectrum of **3** in CD_3OD
Fig. 24S. Product ion scan in positive mode of **4**
Fig. 25S. Product ion scan in negative mode of **4**
Fig. 260S. UV spectrum of **4**
Fig. 27S. IR spectrum of **4** in KBr

Fig. 28S. ^{1}H NMR spectrum (400 MHz) of **4** in CD_3OD

Fig. 29S. ^{13}C NMR spectrum (400 MHz) of **4** in CD_3OD

Fig. 30S. ^{1}H-^{1}H COSY spectrum of **4** in CD_3OD

Fig. 31S. HSQC spectrum of **4** in CD_3OD

Fig. 32S. HMBC spectrum of **4** in CD_3OD

Fig. 1S. Product ion scan in positive mode of **1**

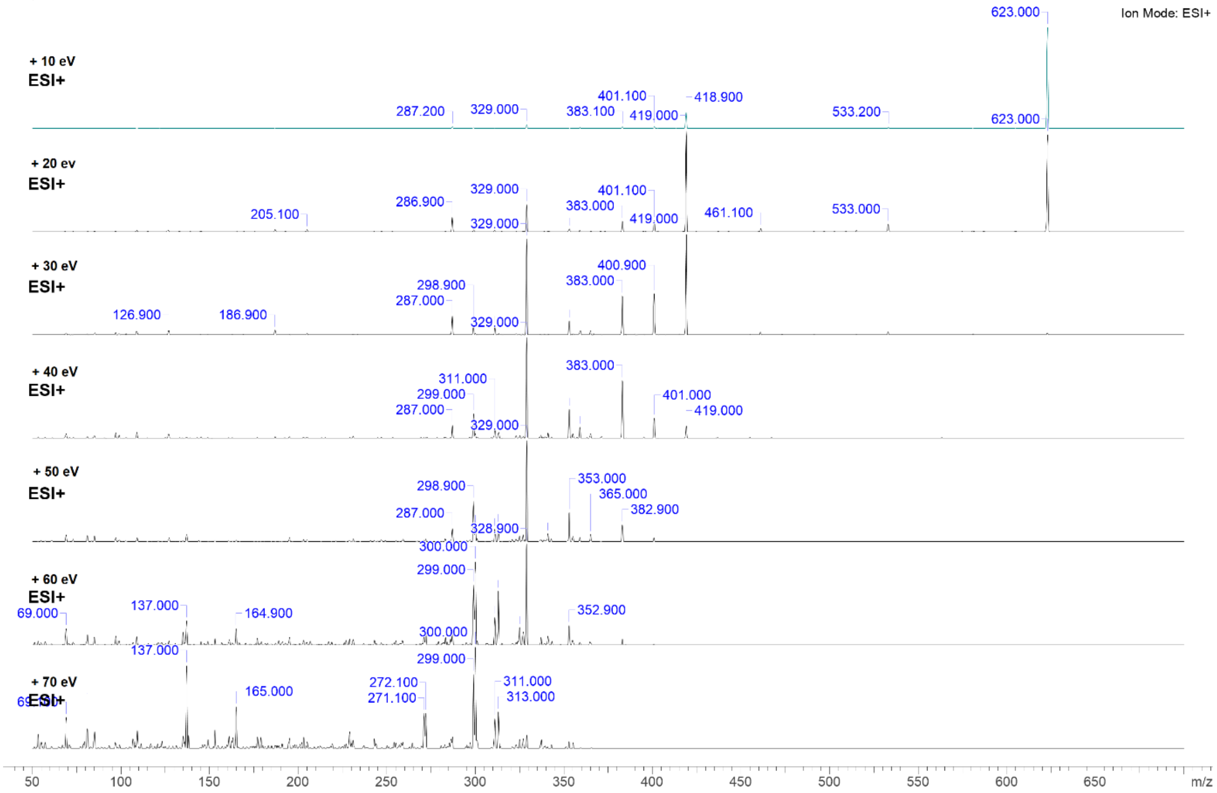

Fig. 2S. Product ion scan in negative mode of **1**

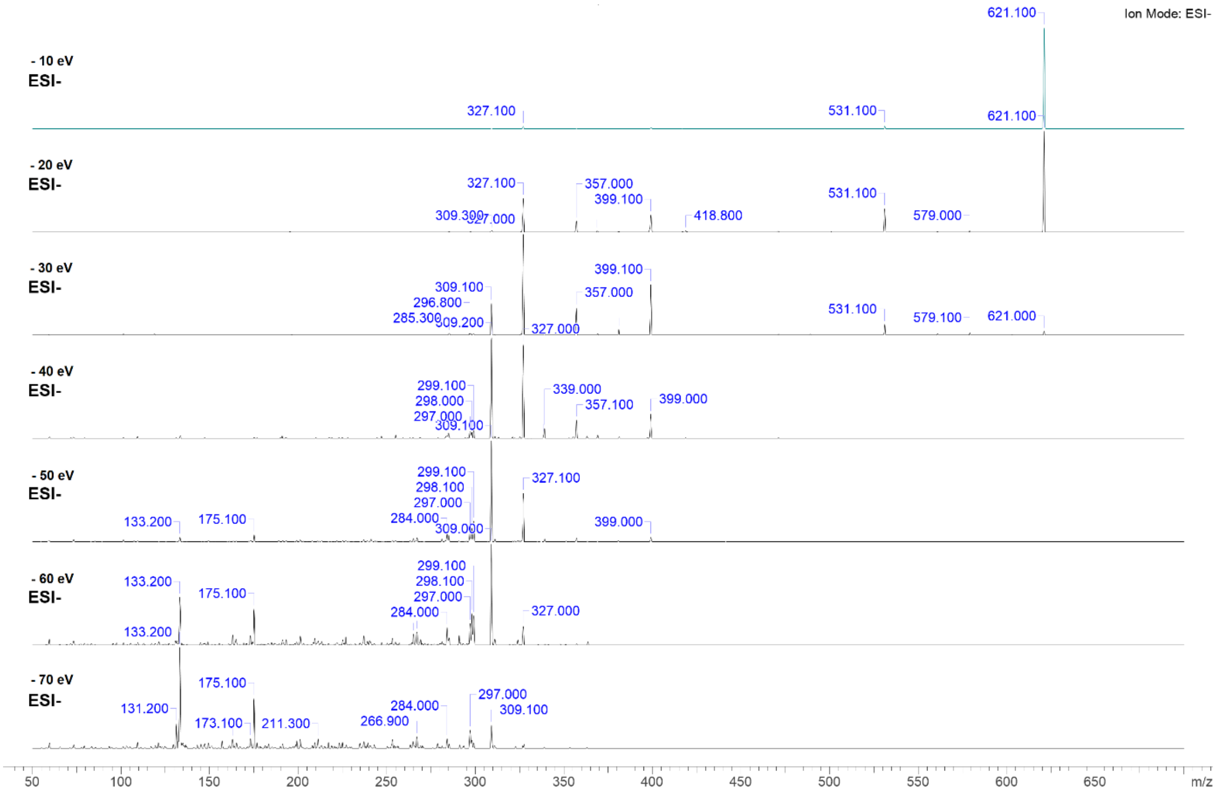

Fig. 3S. UV spectrum of **1**

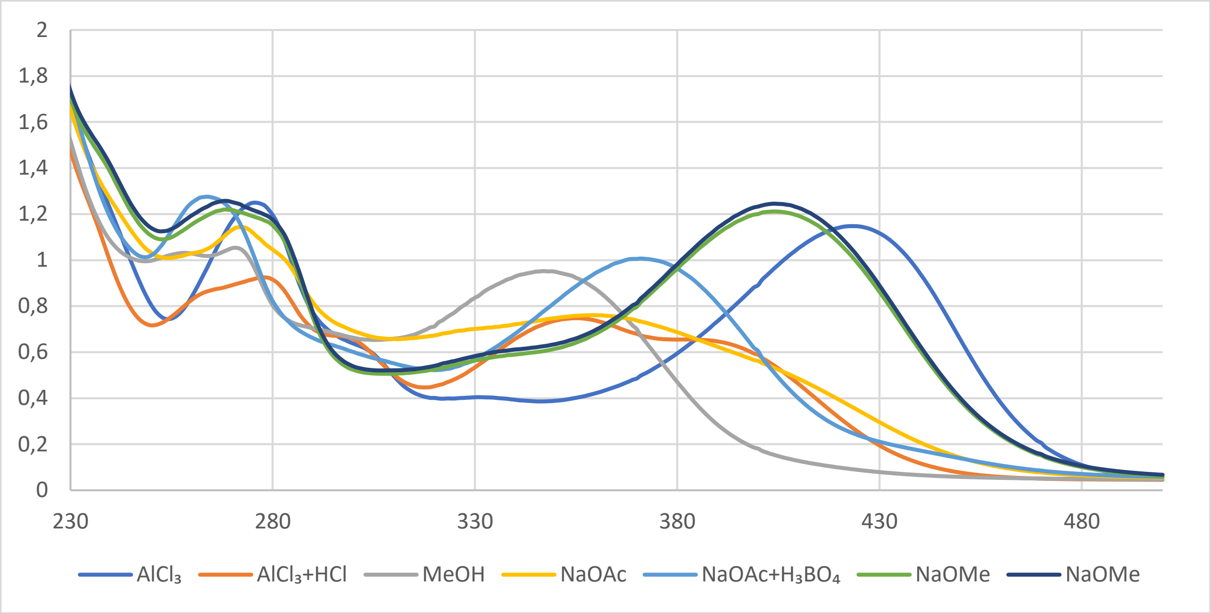

Fig. 3S. IR spectrum of **1** in KBr

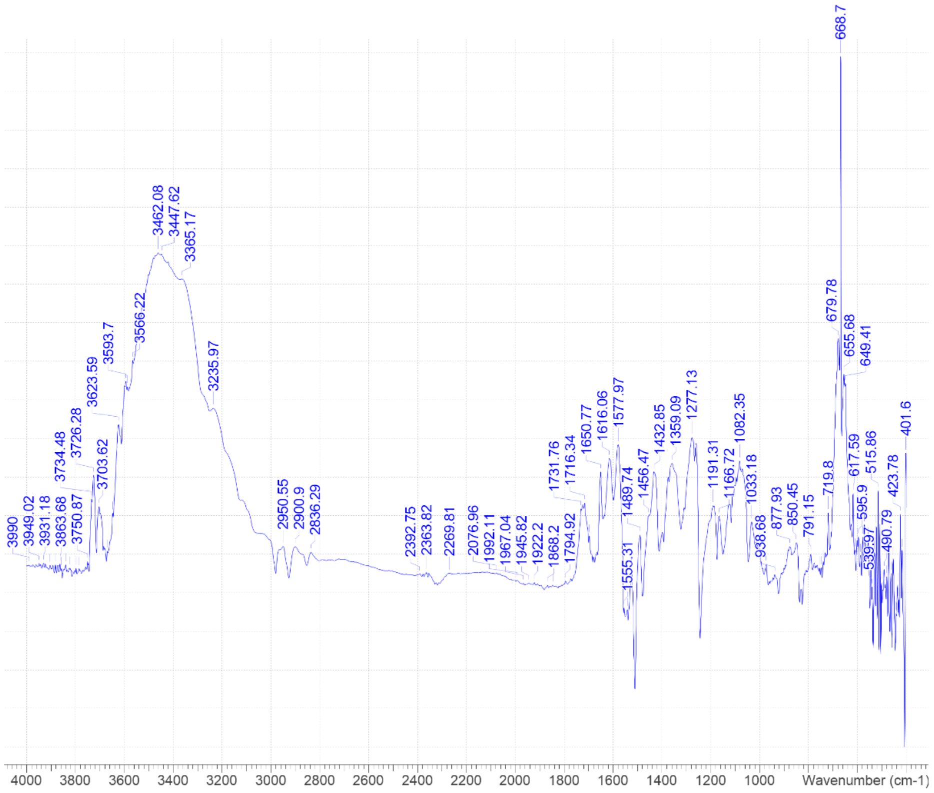

Fig. 5S. ^{1}H NMR spectrum (400 MHz) of **1** in CD_3OD

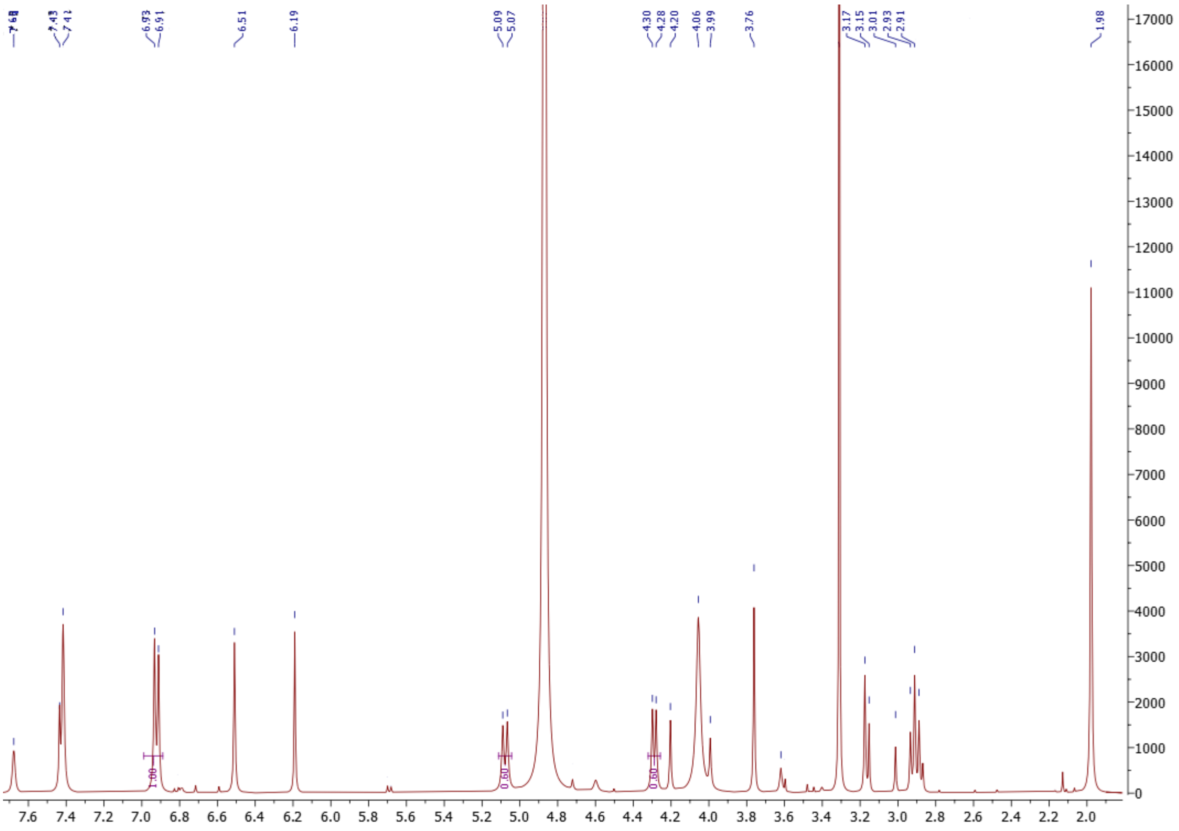

Fig. 6S. ^{13}C NMR spectrum (400 MHz) of **1** in CD_3OD

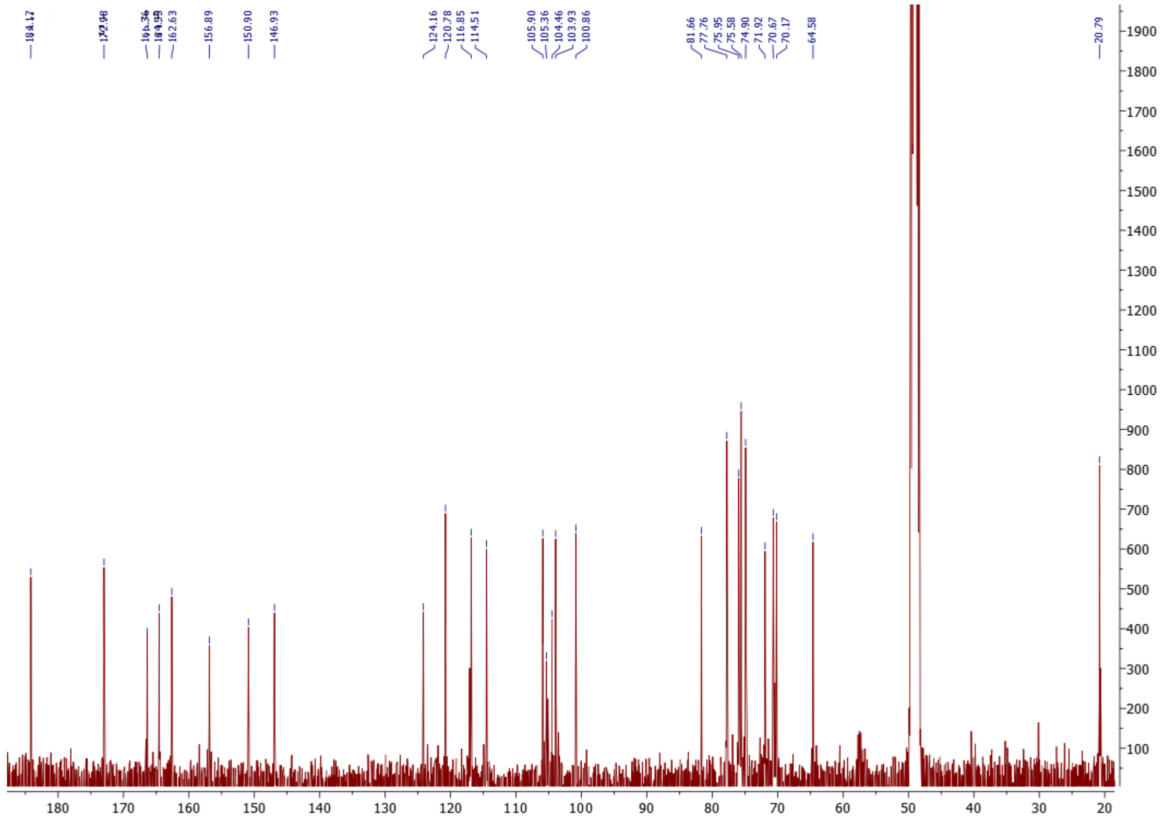

Fig. 7S. ^1H-^1H COSY spectrum of **1** in CD_3OD

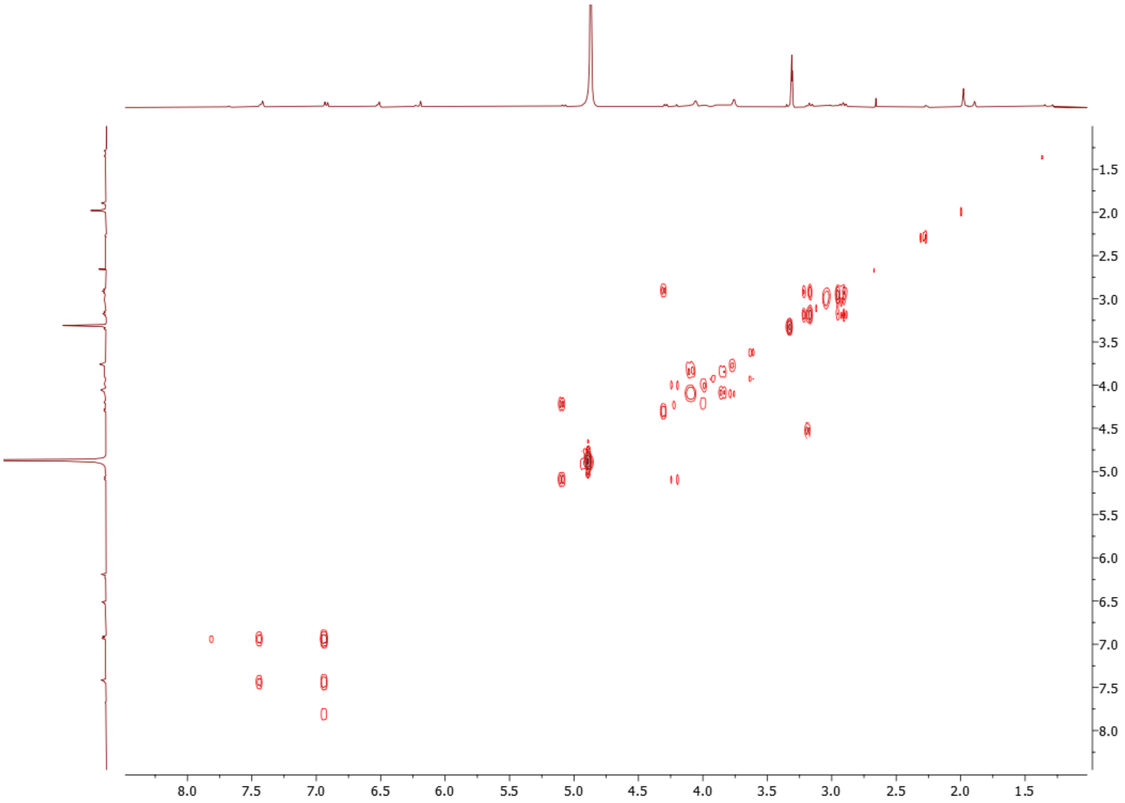

Fig. 8S. HSQC spectrum of **1** in CD_3OD

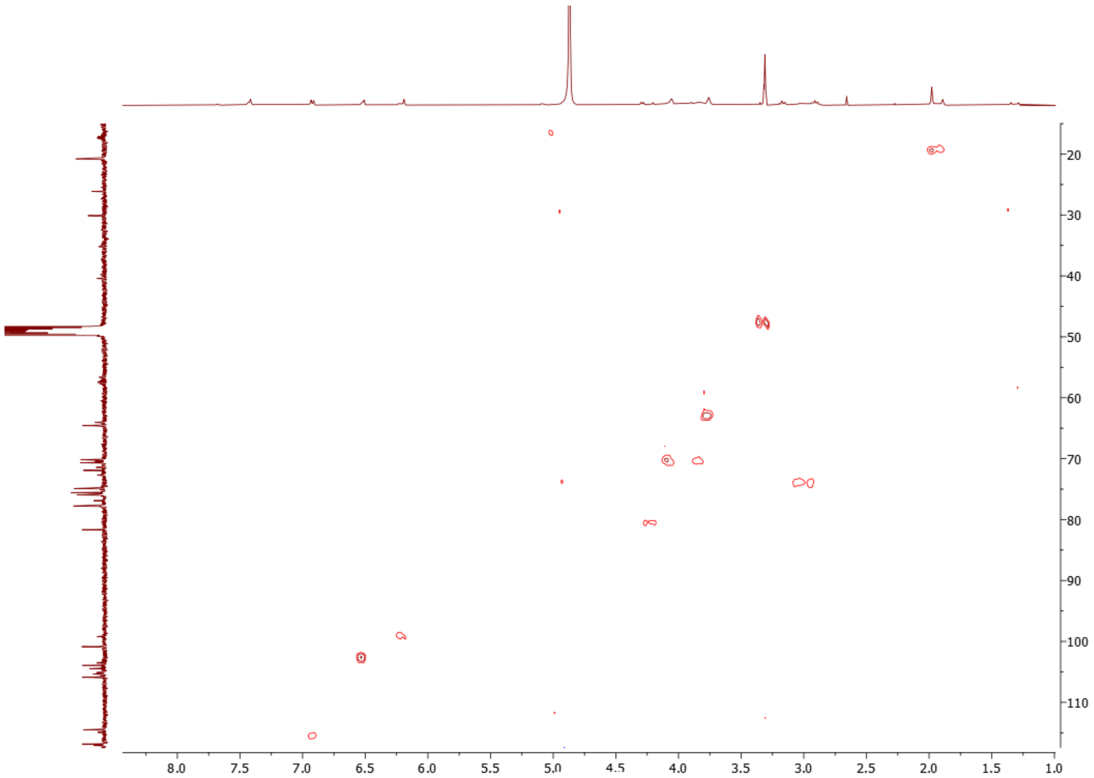

Fig. 9S. HMBC spectrum of **1** in CD_3OD

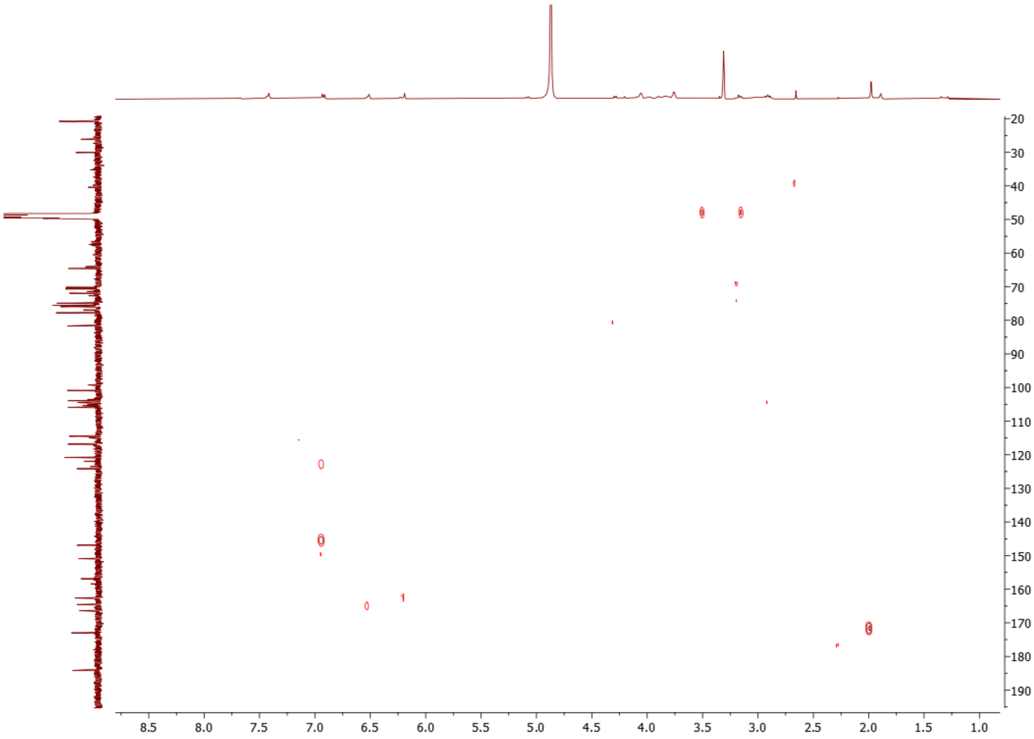

Fig. 10S. Product ion scan in positive mode of **2**

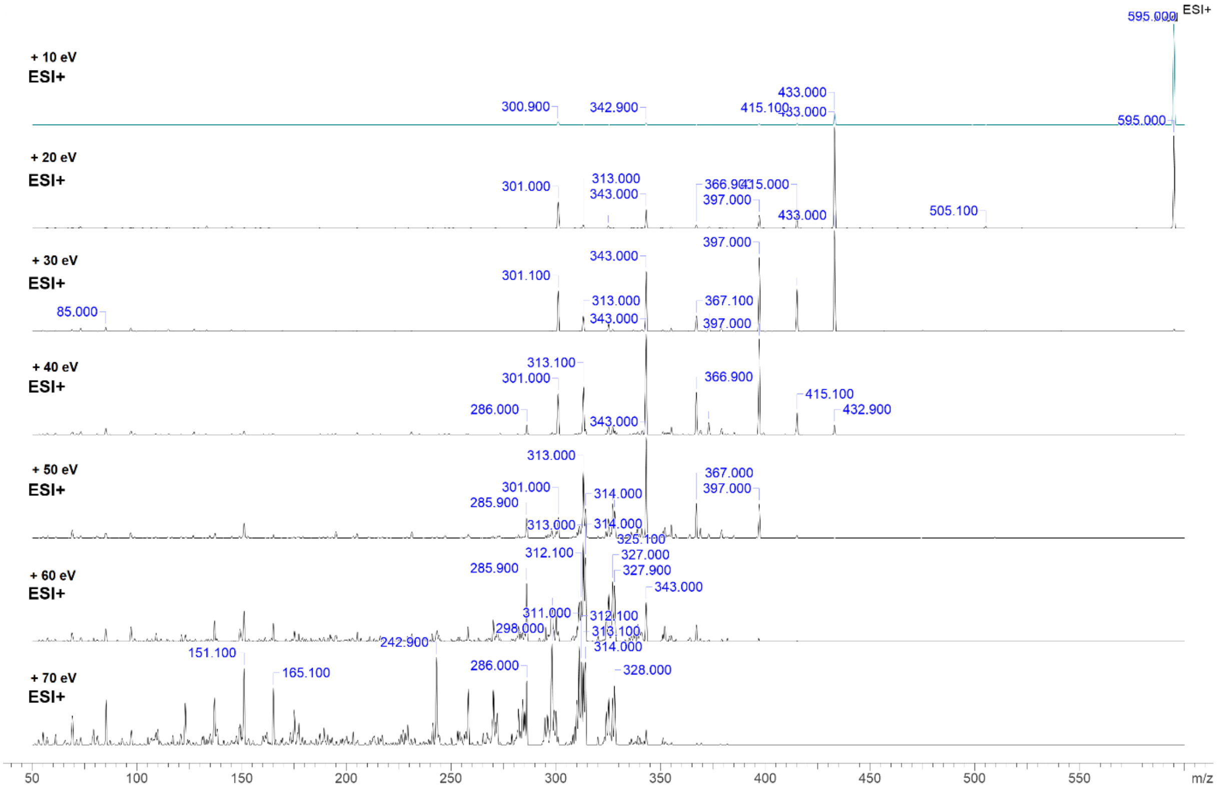

Fig. 11S. Product ion scan in negative mode of **2**

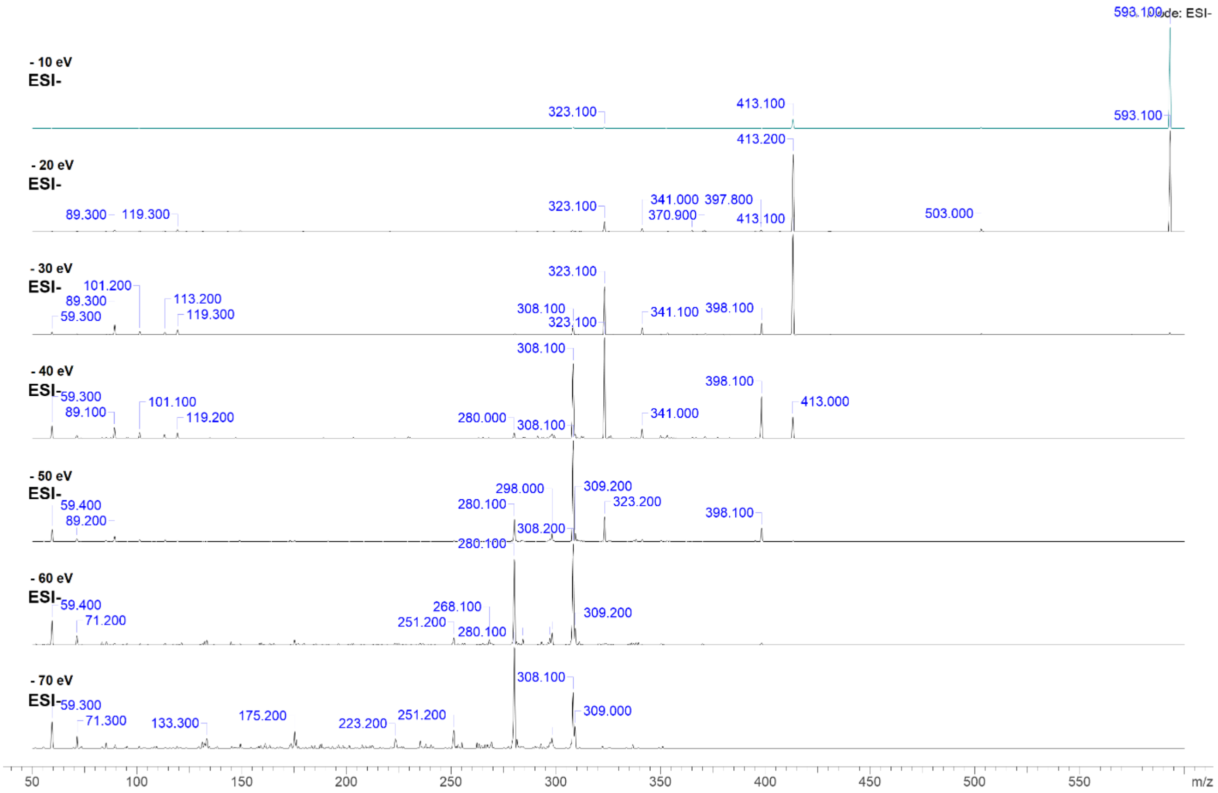

Fig. 12S. UV spectrum of **2**

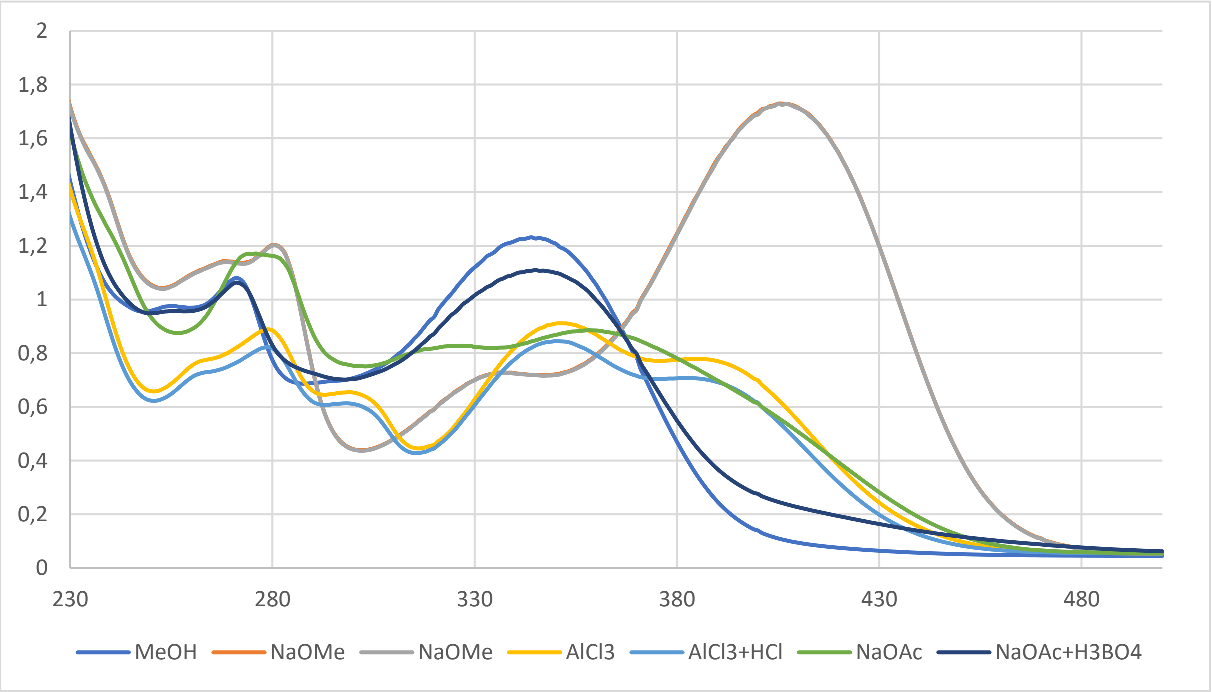

Fig. 13S. IR spectrum of **2** in KBr

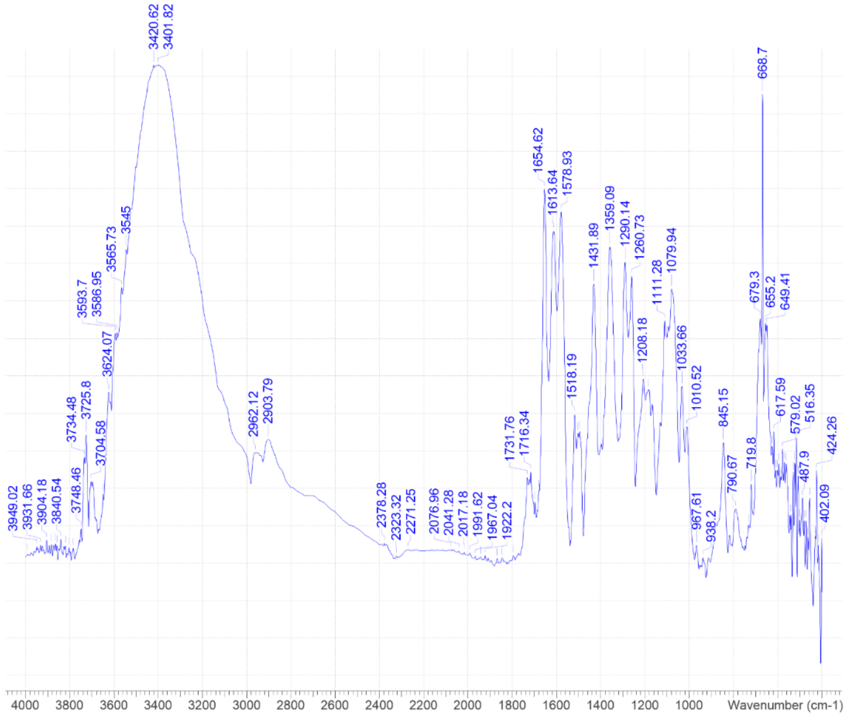

Fig. 14S. ^{1}H NMR spectrum (400 MHz) of **2** in CD_3OD

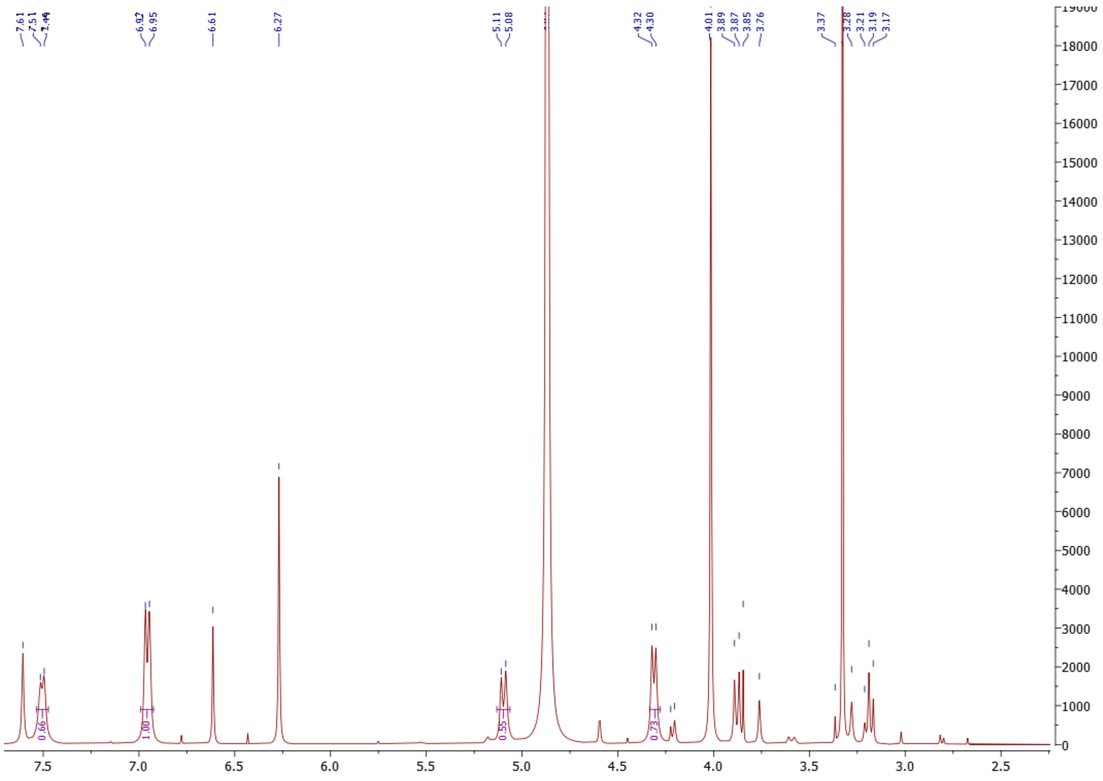

Fig. 15S. ^{13}C NMR spectrum (400 MHz) of **2** in CD_3OD

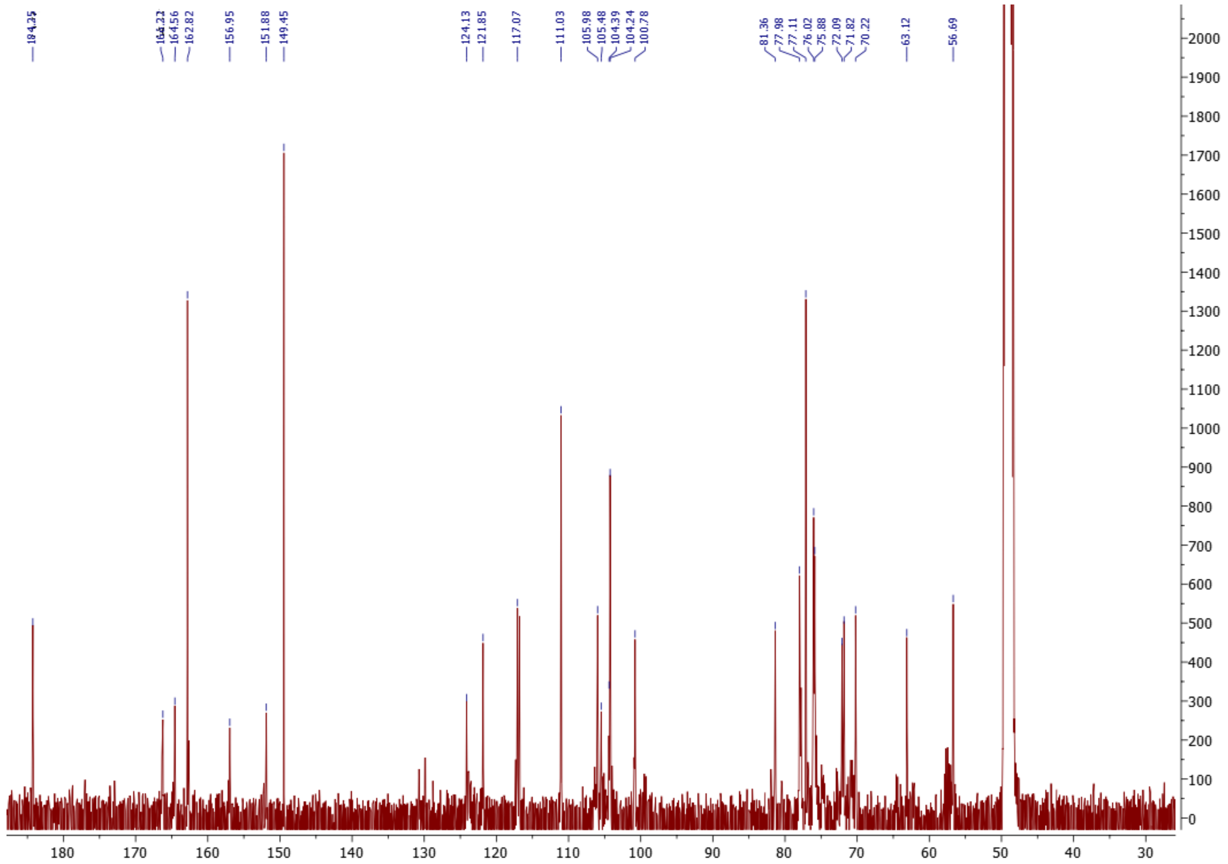

Fig. 16S. ^{1}H-^{1}H COSY spectrum of **2** in CD_3OD

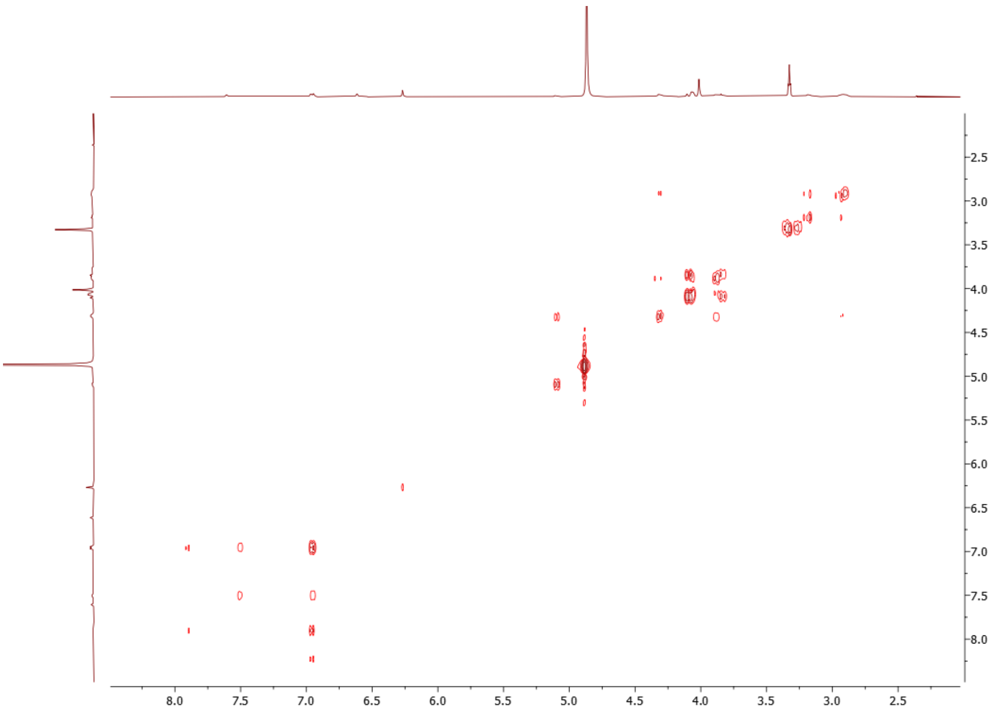

Fig. 17S. HSQC spectrum of **2** in CD_3OD

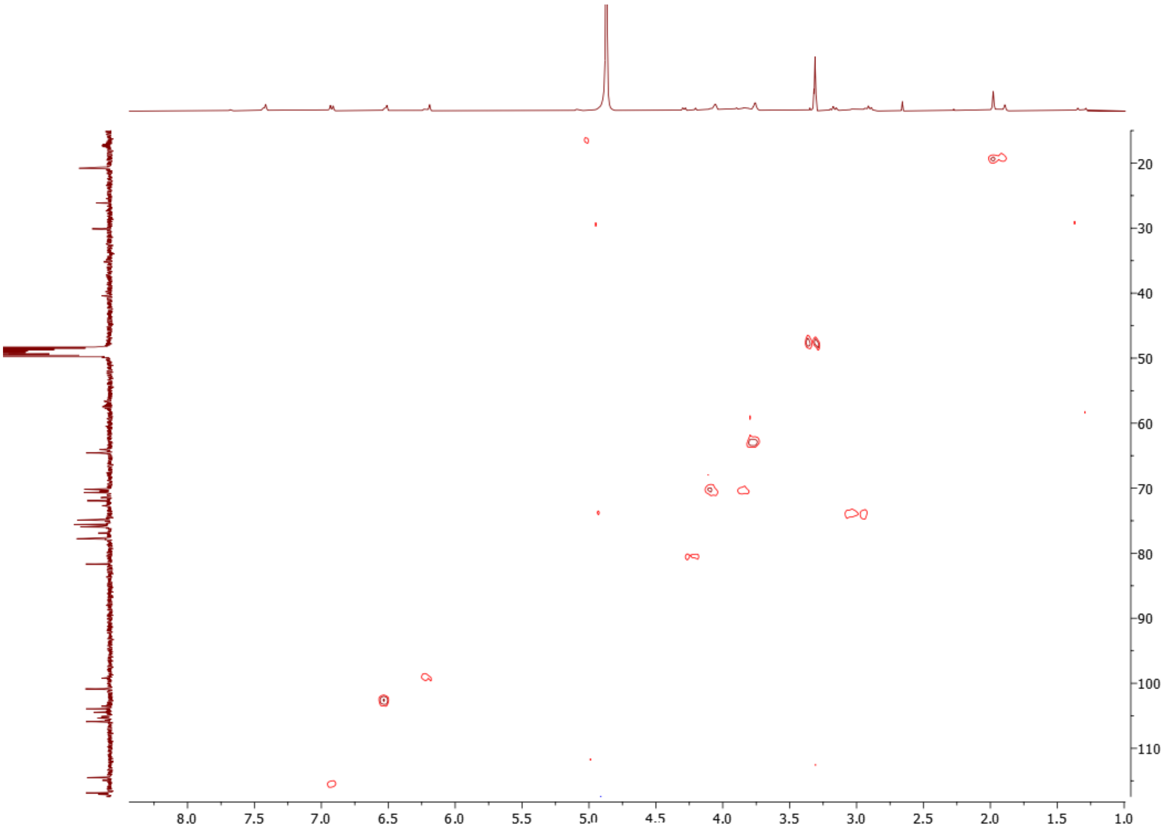

Fig. 18S. HMBC spectrum of **2** in CD_3OD

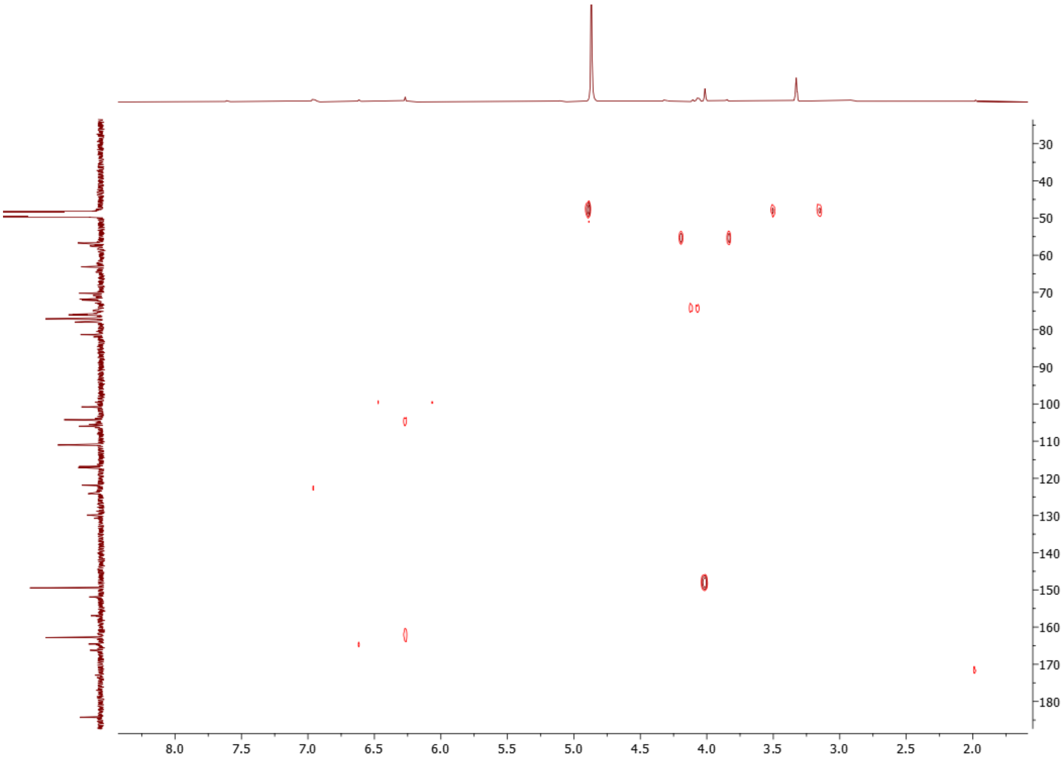

Fig. 19S. ^{1}H NMR spectrum (400 MHz) of **3** in CD_3OD

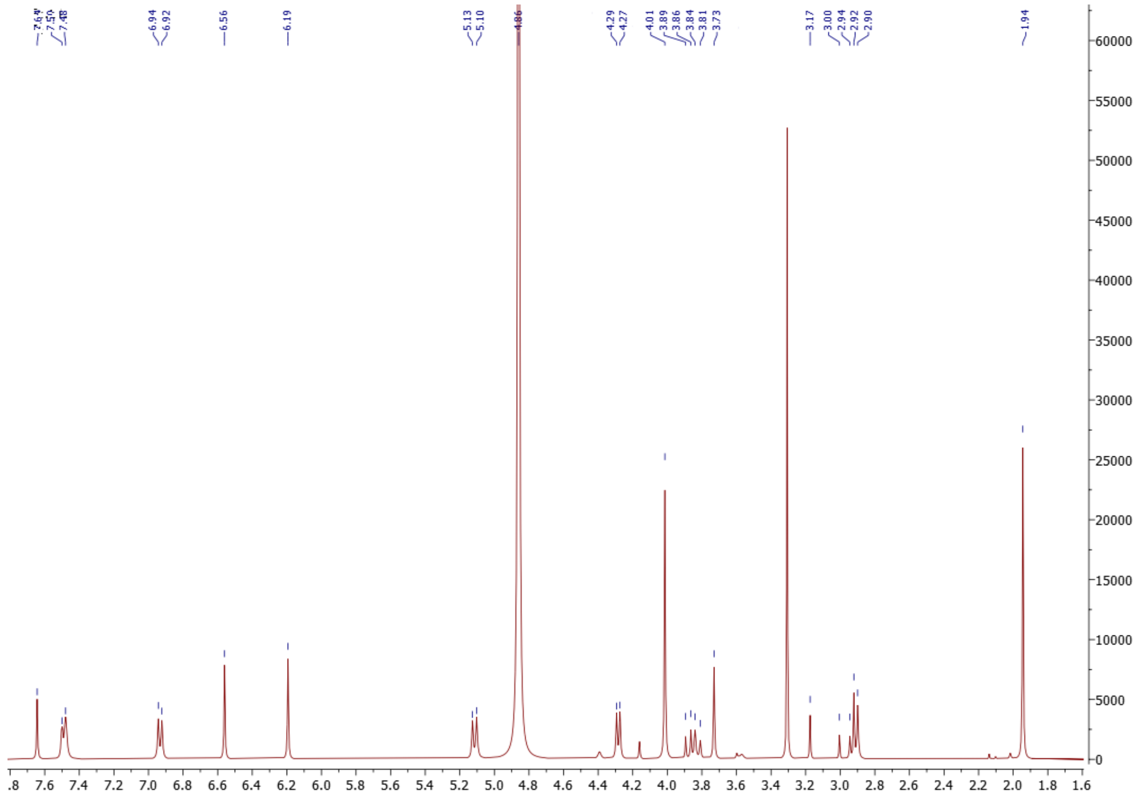

Fig. 20S. ^{13}C NMR spectrum (400 MHz) of **3** in CD_3OD

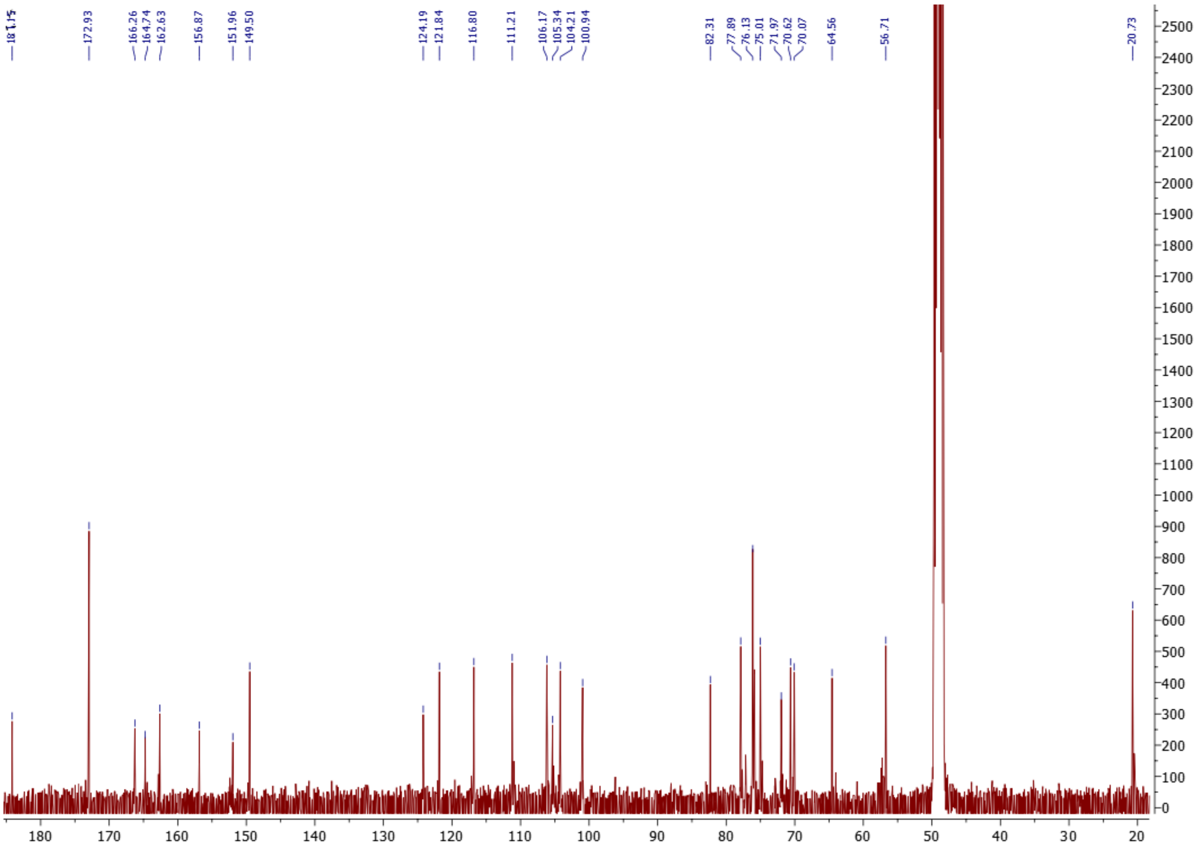

Fig. 21S. ^{1}H-^{1}H COSY spectrum of **3** in CD_3OD

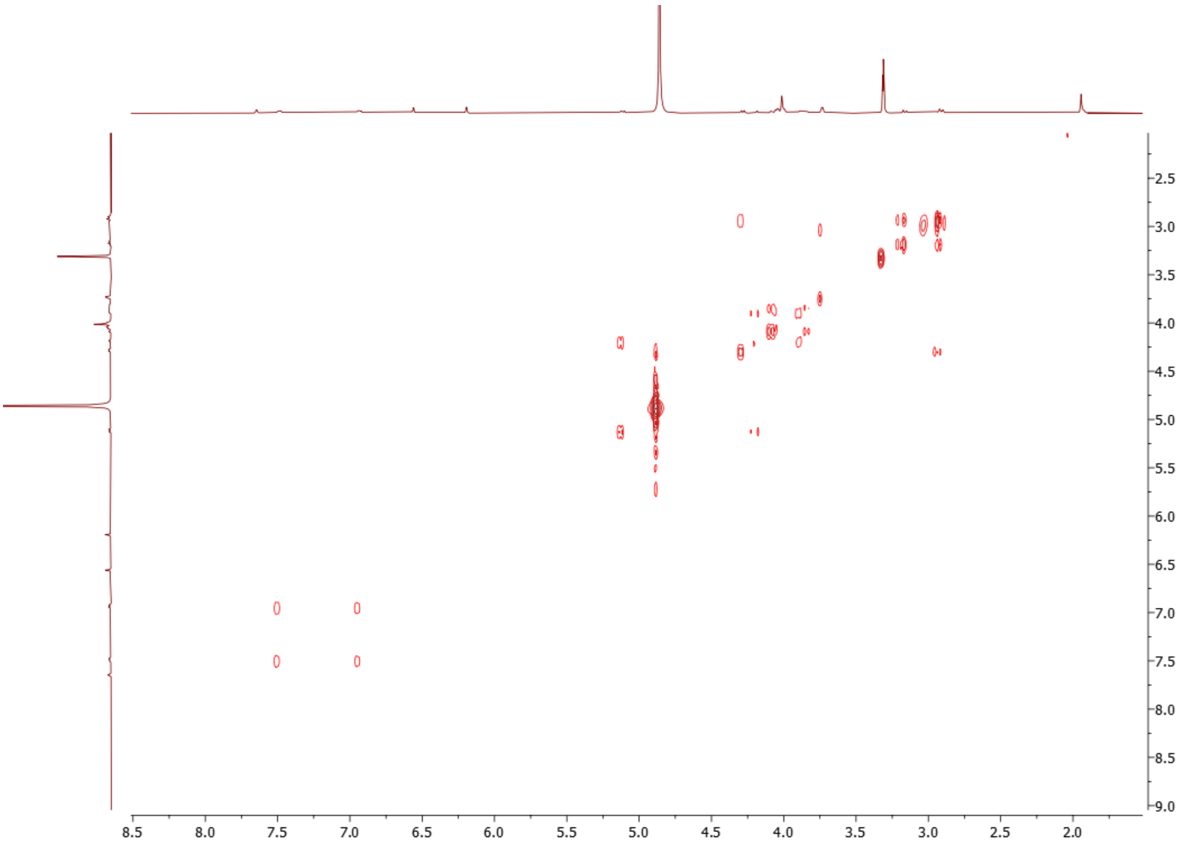

Fig. 22S. HSQC spectrum of **3** in CD_3OD

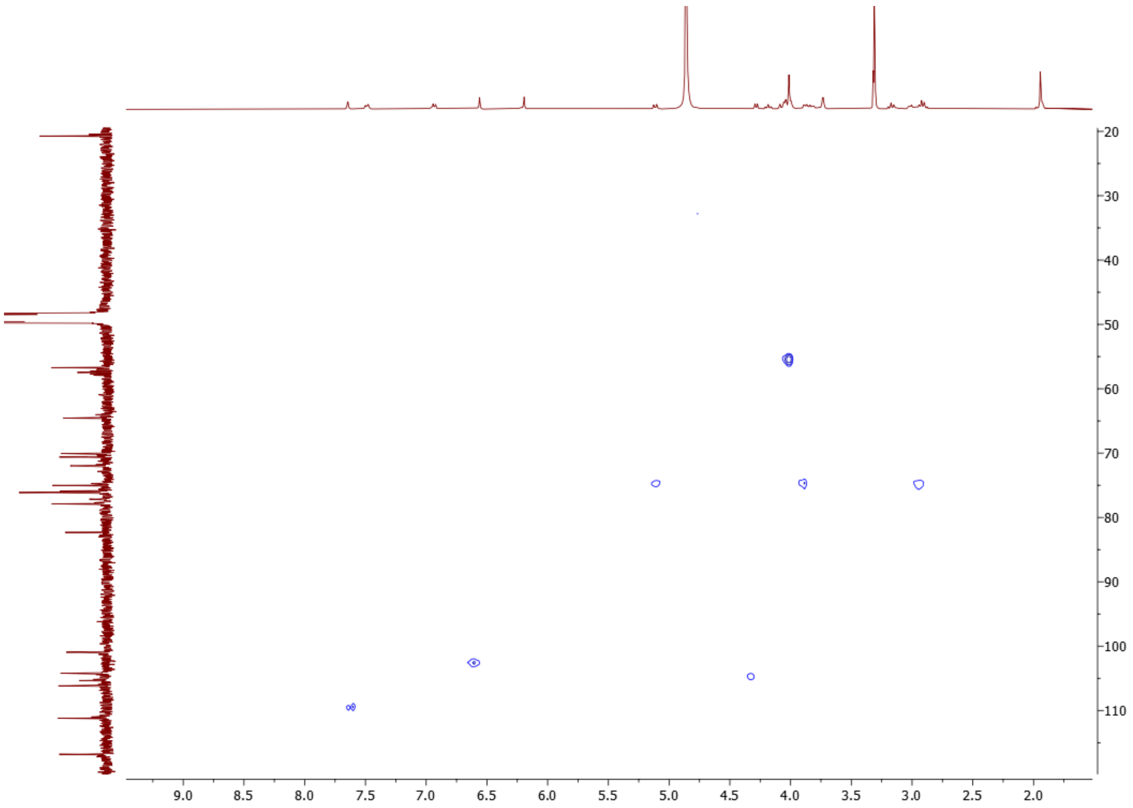

Fig. 23S. HMBC spectrum of **3** in CD3OD

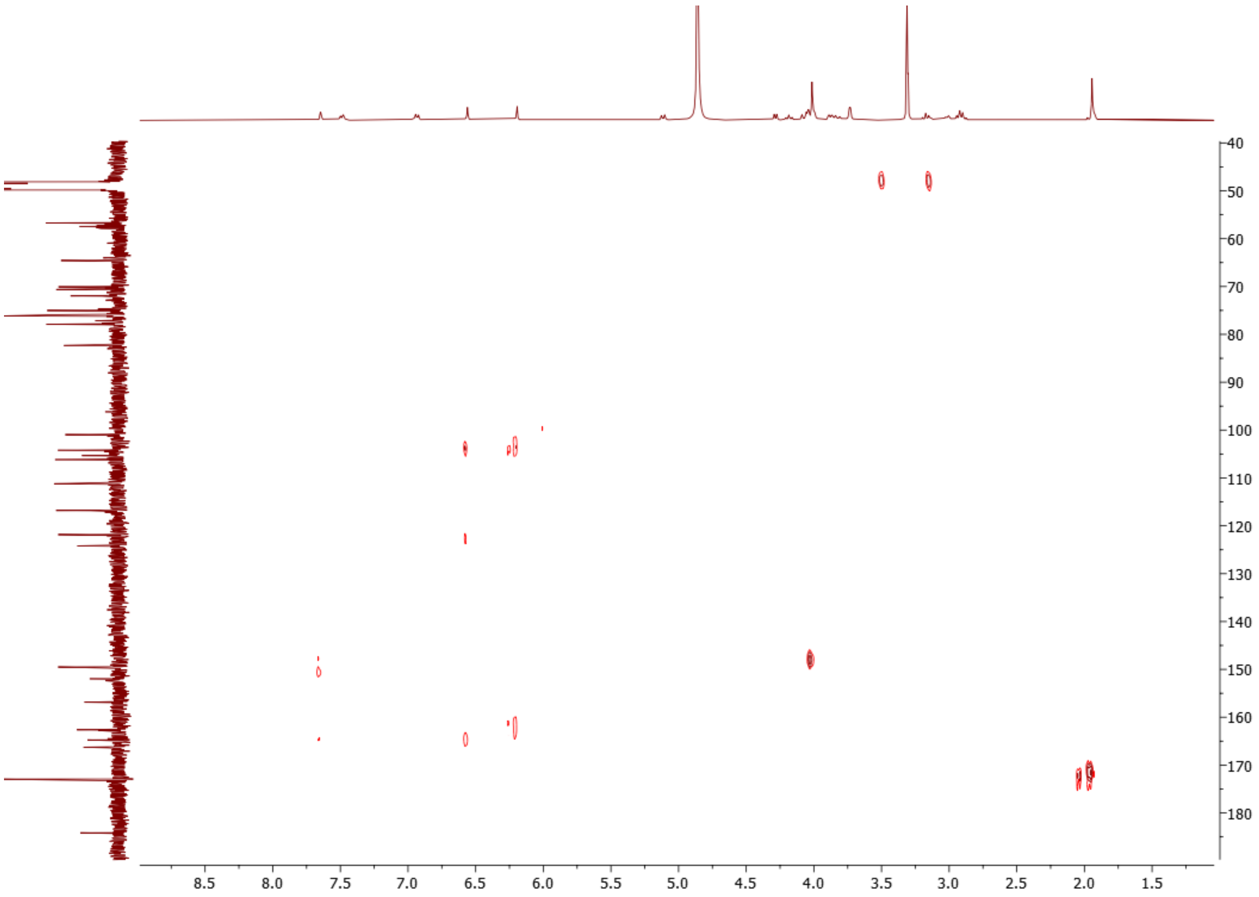

Fig. 24S. Product ion scan in positive mode of **4**

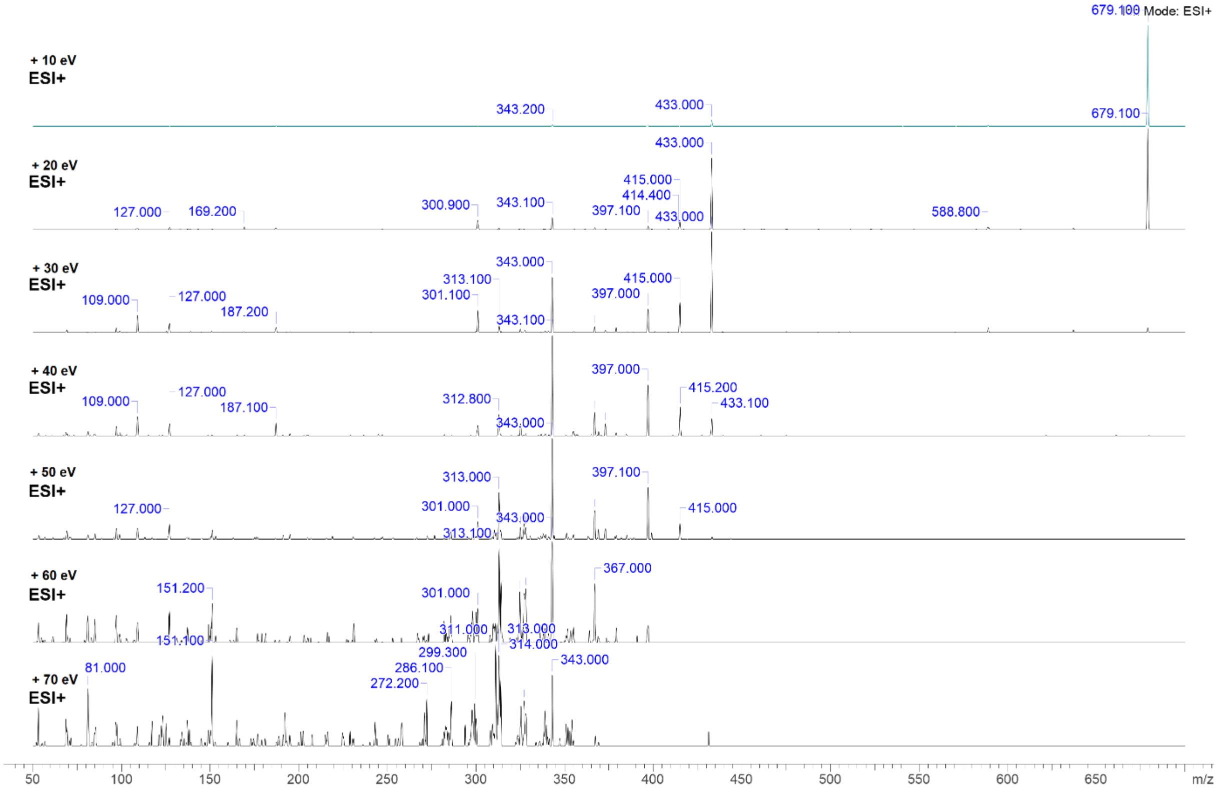

Fig. 25S. Product ion scan in negative mode of **4**

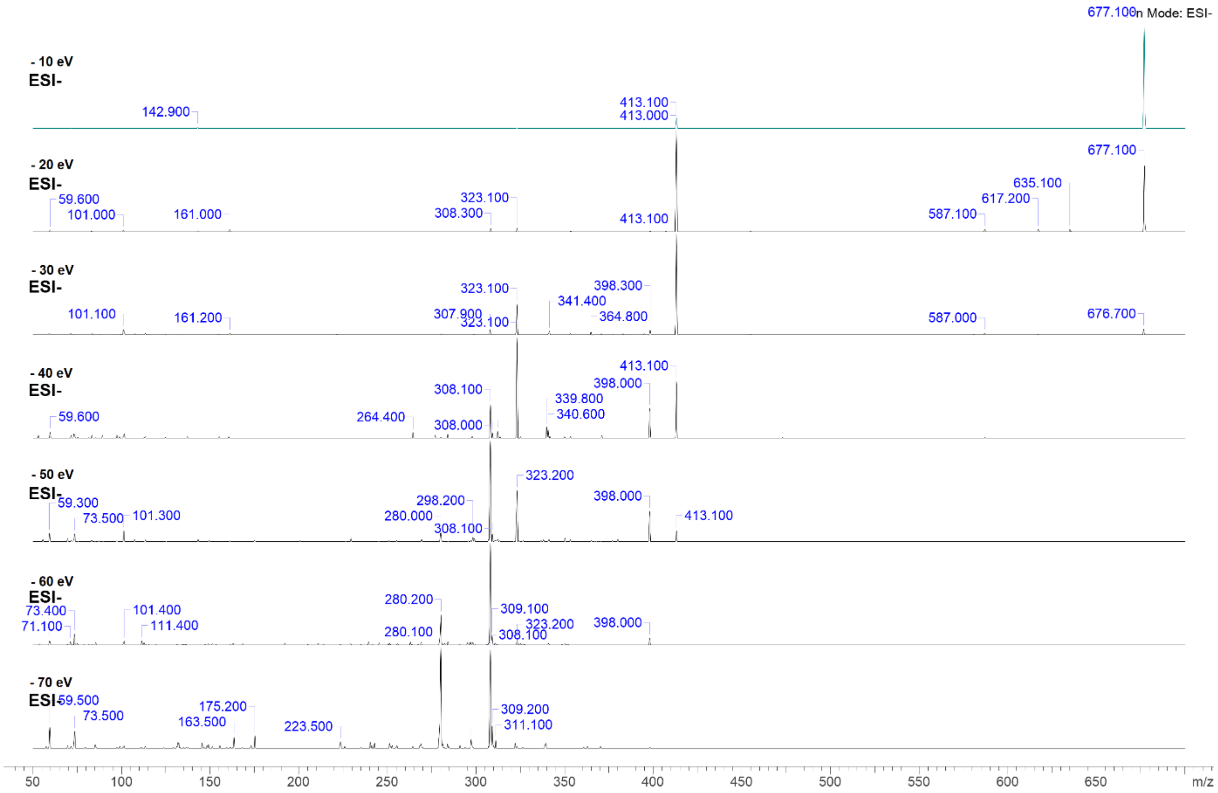

Fig. 26S. UV spectrum of **4**

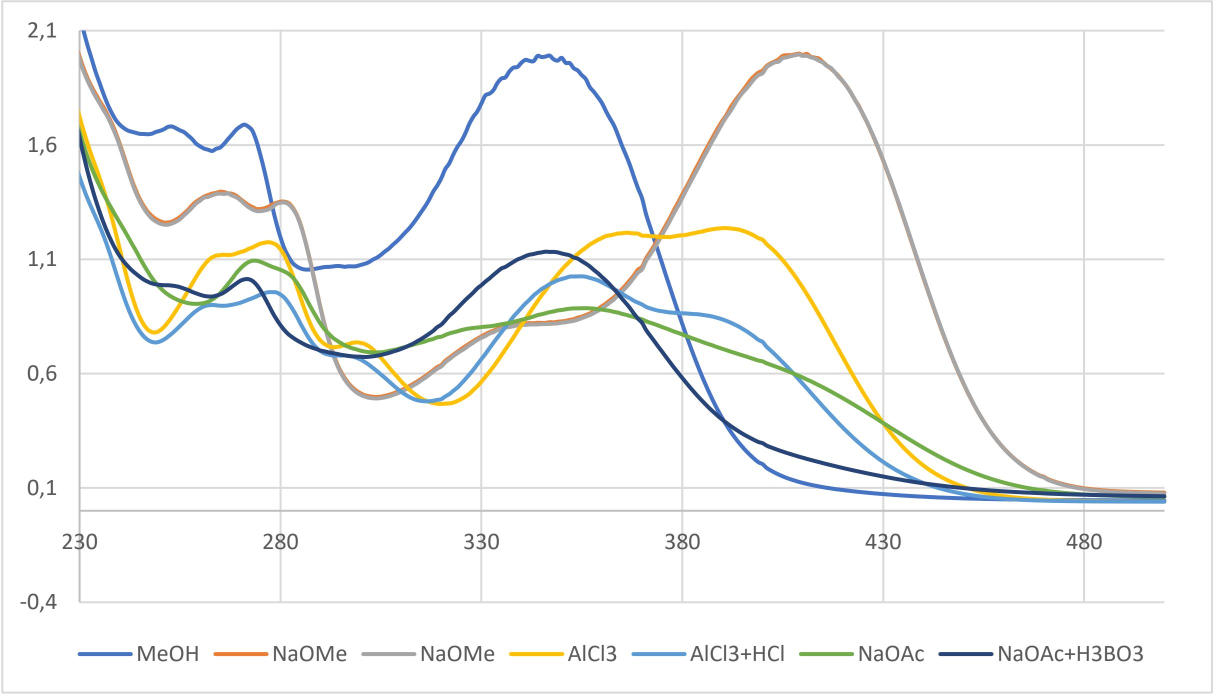

Fig. 27S. IR spectrum of **4** in KBr

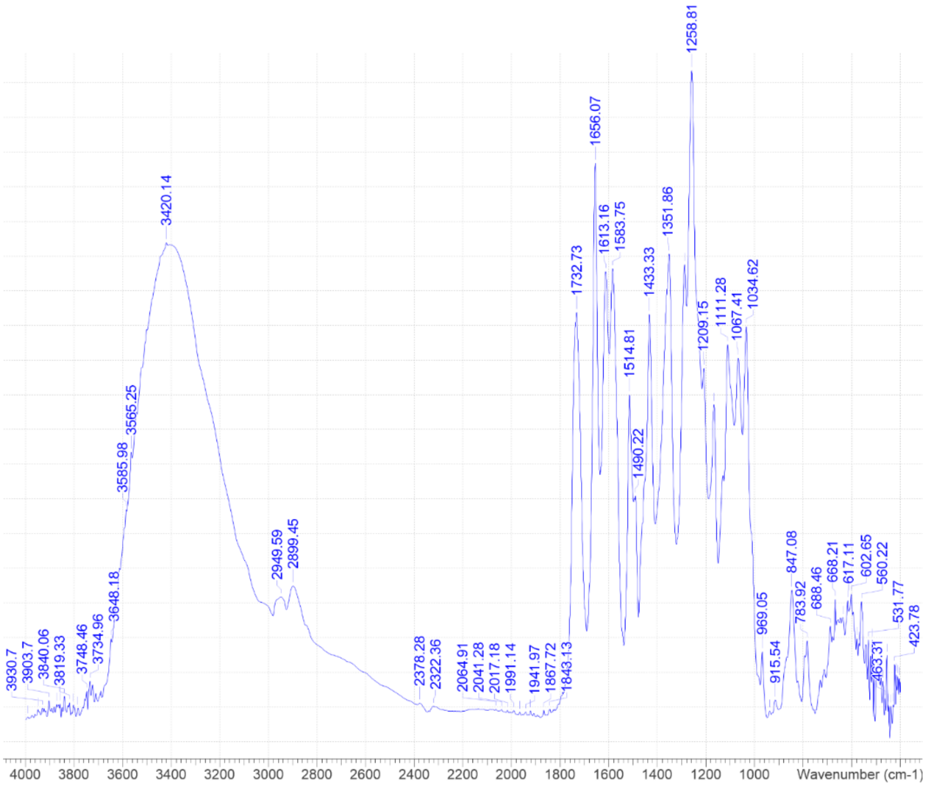

Fig. 28S. ^{1}H NMR spectrum (400 MHz) of **4** in CD_3OD

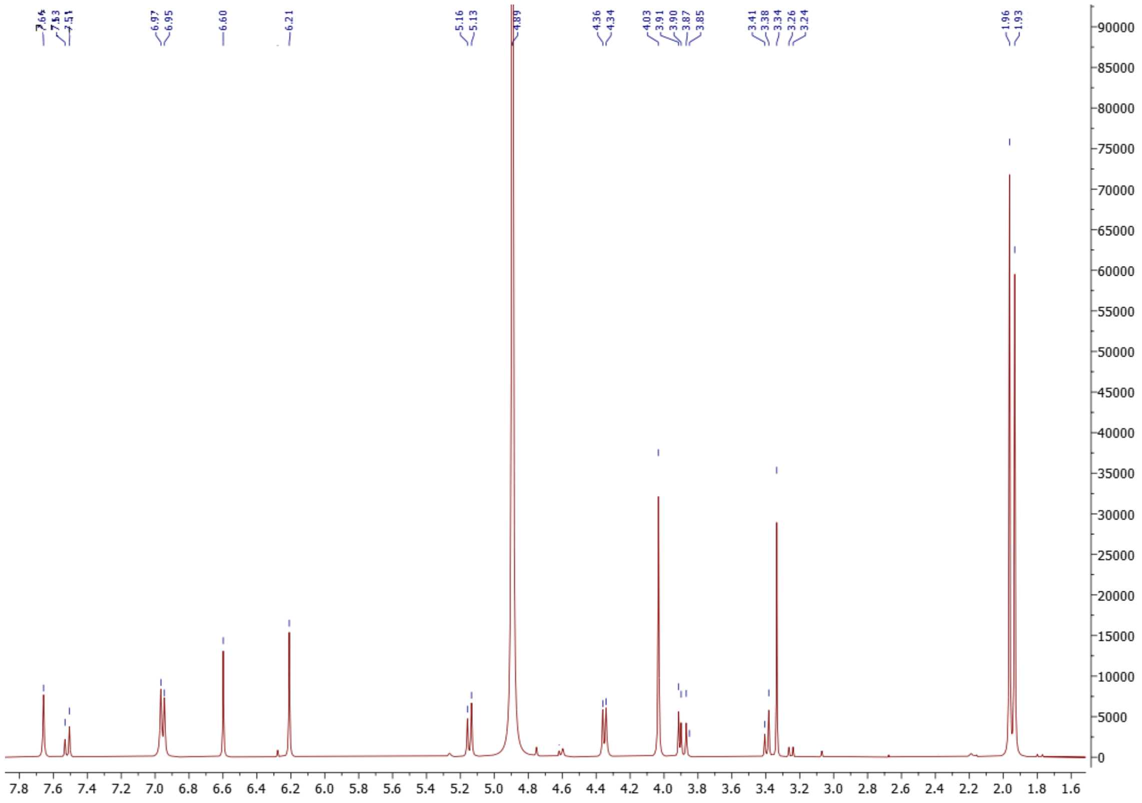

Fig. 29S. ^{13}C NMR spectrum (400 MHz) of **4** in CD_3OD

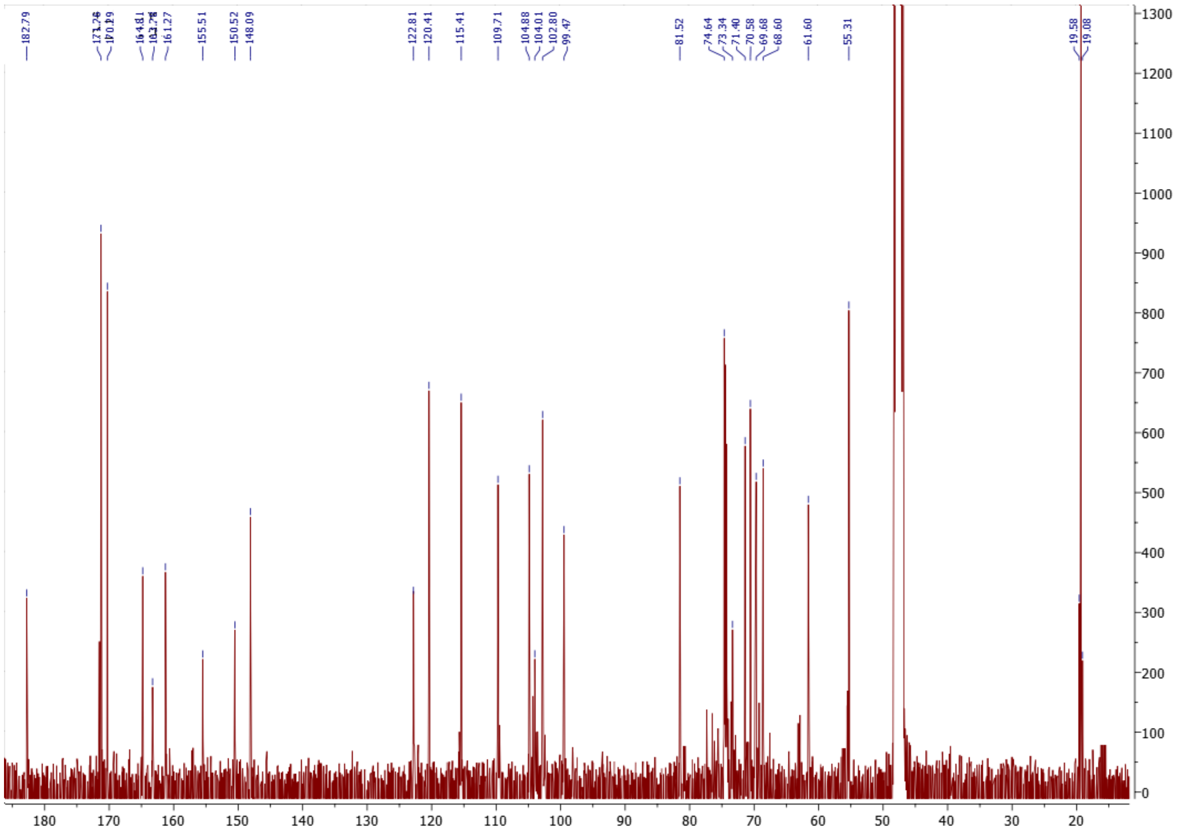

Fig. 30S. ^1H-^1H COSY spectrum of **4** in CD_3OD

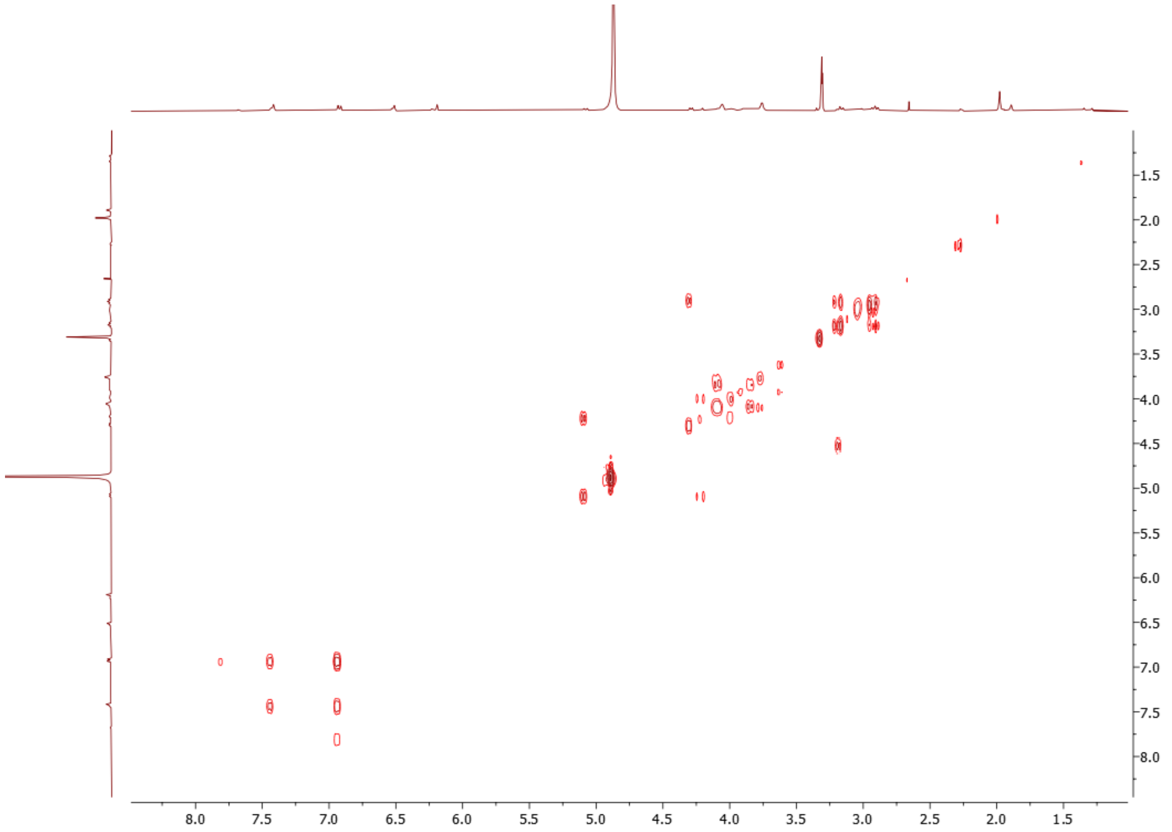

Fig. 31S. HSQC spectrum of **4** in CD_3OD

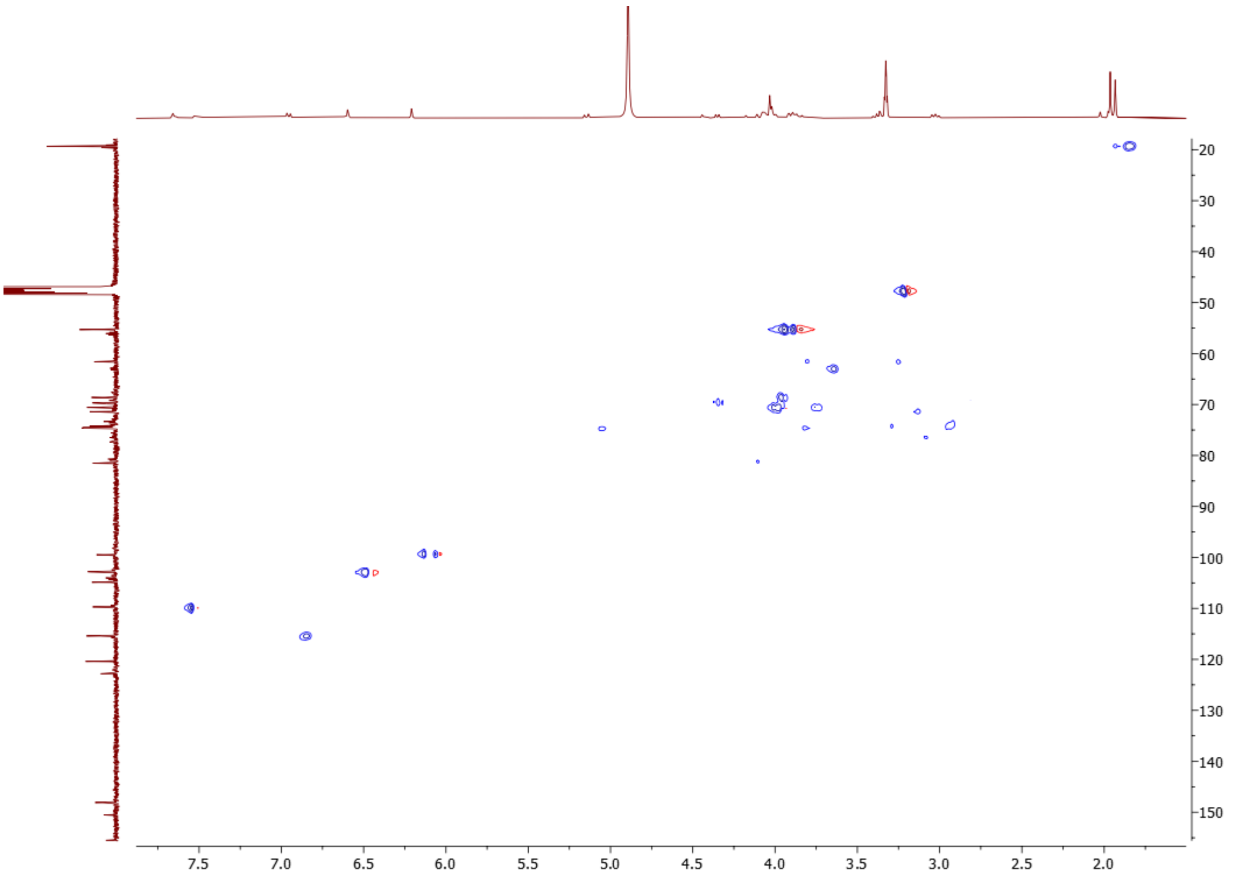

Fig. 32S. HMBC spectrum of **4** in CD_3OD

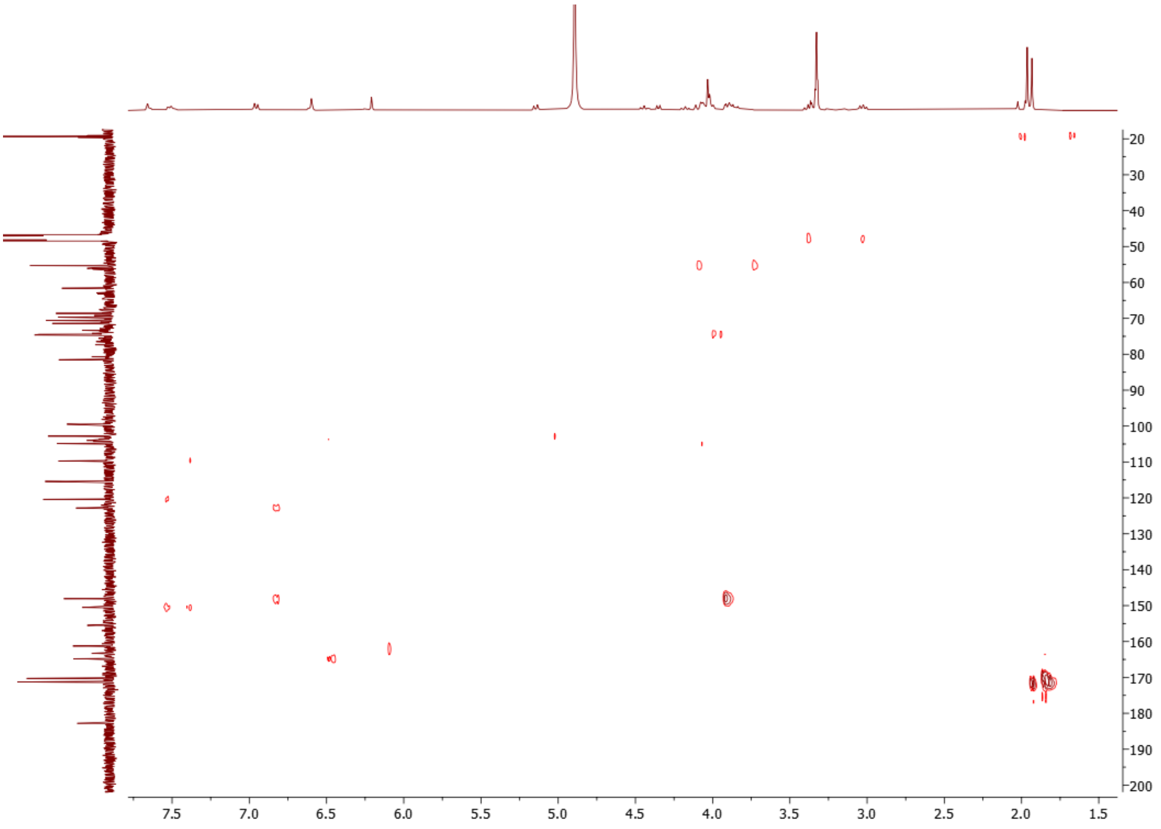

Supplement: Supplementary file 1 [file molecules-26-05631-s001.zip › molecules-1326524-supplementary.pdf]
